# Computational Study of Linker Polarity Effects on Optical Electron Transfer in Imine- and Acylhydrazone-Linked Covalent Organic Frameworks Using Fragment Models

**DOI:** 10.3390/molecules31071179

**Published:** 2026-04-02

**Authors:** Junjin Chen, Dongdong Qi, Jianzhuang Jiang

**Affiliations:** 1 Beijing Key Laboratory for Science and Application of Functional Molecular and Crystalline Materials, Department of Chemistry and Chemical Engineering, School of Chemistry and Biological Engineering, University of Science and Technology Beijing, Beijing 100083, China; chenjunjin08@outlook.com; 2 Guizhou Key Laboratory of Macrocyclic and Supramolecular Chemistry, School of Chemistry and Chemical Engineering, Guizhou University, Guiyang 550025, China

**Keywords:** electron transfer, polarity, photosynthesis, covalent organic framework, DFT, TDDFT

## Abstract

Covalent organic frameworks (COFs) have become a research hotspot in photocatalytic materials in recent years due to their highly ordered structures, tunable topologies, and excellent optoelectronic properties. However, the relationship between linker polarity and the direction of optical electron transfer between adjacent structural units remains poorly understood. This study employs density functional theory (DFT) and time-dependent DFT (TD-DFT) calculations to systematically investigate the effects of polarity reversal in imine and acylhydrazone linkers, as well as different fragment models, on the effective optical net electron transfer. To this end, four representative fragment models (K01–K04) were constructed to simulate linear, multi-connected, and branched environments. The results show that, across all models, the direction of the effective optical net electron transfer from phenyl unit (***Ph***) to ***UnitB*** ($Q_{Ph\to UnitB}$) is highly consistent with the polarity direction of the linker. In imine-linked systems, when the dipole moment of the linker aligns with the intrinsic dipole moment direction between ***Ph*** and ***UnitB***, the absolute value of $Q_{Ph\to UnitB}$ is significantly enhanced; in acylhydrazone-linked systems, only K02 and K03 exhibit similar behavior, while K01 and K04 show no obvious enhancement. These findings provide important guidance for designing efficient photocatalytic COFs: tuning the linker orientation to match the intrinsic polarity of adjacent structural units can significantly improve the efficiency of optical net electron transfer between them.

## 1. Introduction

In recent years, particularly over the past five years, hundreds of photocatalytic materials based on COFs have been designed and synthesized, owing to their well-defined structures and tunable topologies [1,2,3]. For example, in the field of photocatalyzing H_2_O_2_ production, H. Pan and coworkers utilized an acylhydrazone-linked COF to convert H_2_O to H_2_O_2_ and achieved a high efficiency of 10.2 mmol g^−1^ h^−1^ [4]. Y. Chen and coworkers synthesized an imine-linked COF exhibiting a high yield (276 mmol g^−1^ h^−1^) for H_2_O_2_ photosynthesis [5]. Furthermore, Y. Xu and coworkers fabricated ultrathin crystalline amide-functionalized CTF nanosheets and achieved a hydrogen evolution rate of 512.3 μmol g^−1^ h^−1^ of water splitting [6]. By introducing ethynyl groups into the conjugated skeletons of metal-free sp^2^C-COFs to modulate the π-conjugation, Y. Bi and coworkers significantly enhanced the CO_2_ photoreduction performance, achieving a CO production rate of 382.0 µmol g^−1^ h^−1^ [7]. As can be found in the above works, while experimental progress is rapid, a significant research gap remains in the quantitative and systematic prediction of photon/excited electron (*hv*/*e*^−^*) conversion and *e*^−^* transfer details. We gradually realize that *hv*/*e*^−^* conversion and *e*^−^* transfer rules could be systematically summarized and used as the designing strategy for photocatalytic reactions.

Here, we provide one of our typical photocatalytic works [8], which is our first chance to investigate the *hv*/*e*^−^* conversion and *e*^−^* transfer chain in the photocatalytic reactions occurring in COFs. In 2022, we synthesized a special COF, named USTB-11(Cu,Ni), and adopted it as the photocatalytic CO_2_ → CO catalyst, which performed best at the time (conversion rate of 22.1 mmol g^−1^ h^−1^; selectivity of 98%) [8]. Using similar structure units, including tris(*μ*_2_-4-carboxaldehyde-pyrazolato-N,N’)-tricopper (Cu_3_Py_3_), triphenylamine (Ph_3_N), nickel acetate (Ni(OAc)_2_), N’-methyleneformohydrazide (π-linker), and ruthenium bipyridine ([Ru(bpy)_3_]^2+^), we tried several COFs with a different constructing order but found that all of them showed negligible activity in the field of CO_2_ → CO photocatalysts. This confusing case forced us to think about the nature of *hv*/*e*^−^* conversion and the *e*^−^* transfer chain in the viewpoint of electronic structure. In fact, the success of USTB-11(Cu,Ni) was a fortunate coincidence. Without rational design in advance, we fortuitously constructed these five structural units in a proper manner, which formed a smooth energy-reducing chain. This had an energy-conversion/transfer channel starting from the excitation of [Ru(bpy)_3_]^2+^ with the *hv* energy of 2.61 eV and the acceptor Cu_3_Py_3_ with the *e*^−^* energy of 2.55 eV, decreasing to 1.83 eV through the *e*^−^* energy dissipation via the valence cycle of Cu_3_^5+^/Cu_3_^4+^, and continuously decreasing to 1.46 and 1.30 eV via the *e*^−^* energy dissipation hidden in the *e*^−^* transfer chain of Cu_3_Py_3_ → p-linker → Ni(OAc)_2_. At last, via the valence cycle of Ni^2+^/Ni^+^, the energy of *e*^−^* decreases to 1.26 eV, which matches well with the catalysis of CO_2_ → CO. Reviewing the whole chain, we noticed our fortuitous luck, that is, if we dare to switch the position of any two units, this *e*^−^* energy-dissipating chain would be considerably destroyed. To keep this luck, we tried to calculate the orbital energies of structural units in advance, which is used to guide the subsequent COF design and synthesis. In 2023–2025, we gradually completed the designing map of photocatalytic COF materials via the theoretical calculation of light-induced electron flowing modes between typical building blocks, which is the origin of the present work. For example, in 2025, we successfully designed an efficient photon-electron transfer chain embedded in a new COF, EBBT-COF [9]. This new catalyst showed a high H_2_O_2_-producing rate of 5.7 mmol g^−1^ h^−1^, which is the best performance among COF-based photocatalysts reported thus far.

**Scheme 1:**
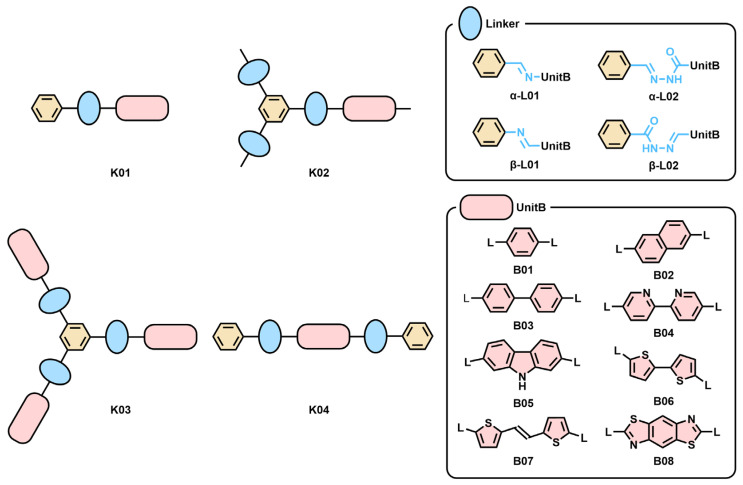
Molecular models used in this study: K01, K02, K03, and K04, including linker orientations (α/β) and representative ***UnitB*** building blocks.

Building upon these successful designs focused on energy-level matching, our investigation has further evolved toward a more fundamental understanding of the electronic factors governing electron transfer. We have identified a critical research gap regarding the role of the linker’s electronic nature—specifically how the dipole moment of asymmetric linkers, such as imine and acylhydrazone, dictates the electron transfer direction. Through a detailed analysis of these optical electron transfer modes, we discovered that the direction and efficiency of cross-linker electron transfer are intrinsically coupled with the orientation of the linker’s polarity. To clarify this relationship in the present work, several typical donor–acceptor (D-A) electron transfer pairs based on imine-linked and acylhydrazone-linked models were constructed based on our donor–linker–acceptor (D-L-A) electron transfer model from the idea of deconstruction of COF skeletons [10,11,12], as shown in Scheme 1. According to the electron transfer performances, several rules are clarified in the following manner:

(1) The direction of the effective net electron transfer from D to A is critically dependent on the orientation of the linker’s dipole moment.
(2) In imine-linked models, when the dipole vector of the linker aligns with the intrinsic D → A polarization direction, the weighted electron transfer is significantly enhanced.

This work tries to demonstrate the controlling law of the electron flowing direction and efficiency, which could be viewed as a designing strategy and selection table for highly efficient photocatalysts based on COFs.

## 2. Results and Discussion

### 2.1. General Theory About hv/e^−^* Behaviors in D-A Linear Models

The D-A model is summarized from the simplest photocatalysts, in which the *hv* is absorbed by a photosensitizer and then converted to *e*^−^* and immediately transfers to the adjacent catalyzing site [13,14].

To begin, it is necessary to place the D-A architecture in the context of photocatalytic material development. For decades, the D-A strategy has served as a cornerstone of molecular photocatalysis, exemplified by the porphyrin-fullerene dyads pioneered and extensively studied by Wasielewsk [15]. More recently, this concept has been extended from isolated molecular dyads to periodic crystalline frameworks; notably, D. Jiang and his coworkers [16] first incorporated the D-A principle into COFs, achieving long-range ordered charge separation with significantly improved efficiency. If this model is moved into a COF skeleton without modification, the *n*-direction connection (*n* ≥ 3) nodes in COFs must only act as structural supporting units rather than catalyzing units, which would be viewed as a type of “serious waste of COF structural units” [5]. But we must firstly discuss it since this is the most basic model in our system, which is used to elucidate our foundation. In this work, the electron transfer mechanism is divided into two mechanisms: without and with photon input.

In the ground state without photon input, the direction and extent of intramolecular charge polarization are primarily characterized by the molecular dipole moment [17,18]. The calculated results indicate that, in most of the systems studied, the center of negative charge is shifted toward the ***UnitB*** moiety, implying that ***UnitB*** tends to act as an electron acceptor, while ***Ph*** tends to act as an electron donor. Table 1 presents the total dipole moment magnitude (in Debye) and the projection of the dipole moment vector along the ***Ph*** → ***UnitB*** direction for each ***Ph***-***UnitB*** system. The results show that the dipole projections for B01, B02, and B03 are close to zero or slightly positive, whereas those for B04, B05, B06, B07, and B08 are distinctly negative, ranging from −0.08 to −1.22 D. These demonstrate that the ground-state polarization is very weak in the first three systems (primarily hydrocarbon-based aromatic units) but becomes significantly stronger in the latter five systems (containing N, S, or other heteroatoms). Overall, across these ***Ph***-***UnitB*** systems, ***Ph*** exhibits donor-like character, while ***UnitB*** shows acceptor-like character, with this trend being particularly pronounced in the heteroatom-containing ***UnitB*** units.

**Figure 1:**
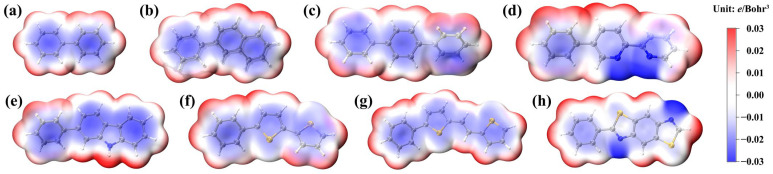
Molecular electrostatic potential (ESP) maps of ***Ph***-***UnitB*** systems (**a**–**h**), where ***UnitB*** corresponds to B01–B08, respectively. The ESP is mapped onto the 0.001 a.u. electron density isosurface. Color scale: −0.03 (blue) to 0.03 (red) $e/{\mathrm{Bohr}}^{3}$. Atom colors: gray, carbon; white, hydrogen; blue, nitrogen; yellow, sulfur.

To gain a deeper understanding of these polarization trends, we analyzed the molecular electrostatic potential (ESP) maps, Figure 1. The ESP maps provide visual evidence that aligns with the dipole moment analysis and offers deeper insights into the electronic distribution. For the hydrocarbon-based aromatic systems (B01–B03), the positive ESP regions are primarily located on the hydrogen atoms, surrounding the molecule, while the negative regions are distributed above and below the carbon atoms. This highly symmetric distribution leads to a near-complete overlap of the positive and negative charge centers, resulting in the negligible dipole moments. In contrast, the introduction of heteroatoms significantly alters the ESP distribution. For the sulfur-containing systems (B06 and B08), the S atoms exhibit distinct negative ESP regions. In the nitrogen-containing systems (B04, B05, and B08), the N atoms possess intense negative ESP values, indicating strong electron-withdrawing character. Notably, for B04 and B05, the N atoms are positioned such that the negative ESP is concentrated perpendicular to the ***Ph***-***UnitB*** axis, leading to a large overall magnitude but a smaller projection along the ***Ph*** → ***UnitB*** direction. Conversely, for B08, the orientation of the heteroatoms enhances the projection along the molecular axis. Overall, these ESP distributions confirm that ***Ph*** acts as a donor while the heteroatom-containing ***UnitB*** moieties serve as potent acceptors.

If *hv* is introduced into this catalyzing system, it will evoke a photosensitizing unit, which acts as the startpoint of the *hv*/*e*^−^* converting/transferring process [19,20]. This *hv* would induce a typical *e*^−^* transferring process rather than the aforementioned *e*^−^ transferring process at the ground state [21,22]. In fact, this design is the main designing aim of the photocatalyzing field [23]. As can be found in Table S1, we calculated all the ***Ph***-***UnitB*** pairs under typical absorbing wavelengths for the K01 model. The usage of Table S1 is a little complex that users must firstly determine one factor, that is, the *hv* wavelength. For example, if readers are designing a new COF photocatalyzing material, which is scheduled to use the light with the wavelength of 300 nm, you can select the benzene-C=N-benzene pair (300.8 nm), Naphthalene(Z)-N=C-benzene pair (309.8 nm), Naphthalene(S)-C=N-benzene pair (303.6 nm), and biphenyl-N=C-benzene pair (306.0 nm) in Table S1. Next, you should check the net electron transfer from the ***Ph*** to ***UnitB*** ($\Delta q_{Ph\to UnitB}$) column and select the largest (or proper) one. After your selection, you can directly get the electron flowing direction between ***Ph*** and ***UnitB***, where a positive value denotes a net electron flow from ***Ph*** to ***UnitB***, and a negative value indicates the reverse. In addition, you can use Table S1 in another manner, that is, if you have selected a specific ***UnitB*** (e.g., Naphthalene), then you select a linker according to the $\Delta q_{Ph\to UnitB}$ column, for which the value of the electron transfer amount column is proper. Please pay attention as, due to the change in the *hv* wavelength, the *e*^−^* behaviors would be completely different. For example, for benzene-C=N-2,2′-Bipyridine(S) described in Table 2 and Table S1, when the *hv* exciting wavelength is 172.6 or 171.1 nm, the $\Delta q_{Ph\to UnitB}$ are positive values, indicating the electron flowing direction of ***Ph***(donor) → ***UnitB***(2,2′-Bipyridine(S), acceptor). In contrast, when the *hv* exciting wavelength is 257.7, 180.0, or 172.6 nm, the $\Delta q_{Ph\to UnitB}$ are negative values, indicating the electron flowing direction of ***Ph***(acceptor) ← ***UnitB***(2,2′-Bipyridine(S), donor). In one word, the donor/acceptor pair is not fixed in the photocatalyzing reactions.

In addition, a special discussion on “how the change in excitation wavelength affects the direction of electron transmission” is obviously valuable. The direction and magnitude of electron transfer (represented by $\Delta q_{Ph\to UnitB}$) are inherently determined by the spatial redistribution of electrons from the ground-state occupied orbitals to the excited-state unoccupied orbitals.

**Figure 2:**
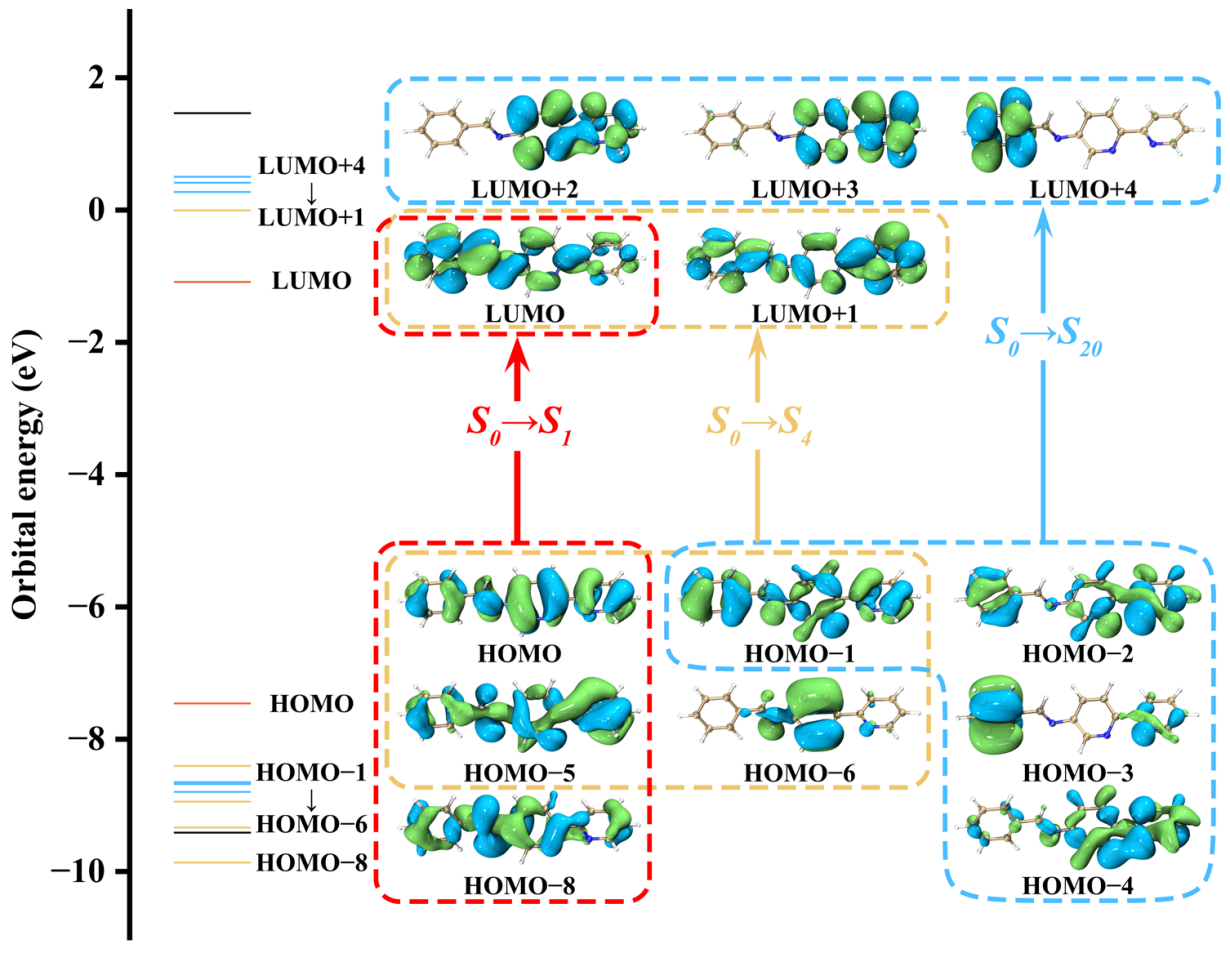
Dominant molecular orbital contributions and energy levels for electronic transitions ($S_{0}\to S_{1}$, $S_{0}\to S_{4}$, and $S_{0}\to S_{20}$) of benzene-C=N-2,2′-Bipyridine(S). The orbital isovalue is set to 0.025 a.u.

**Figure 3:**
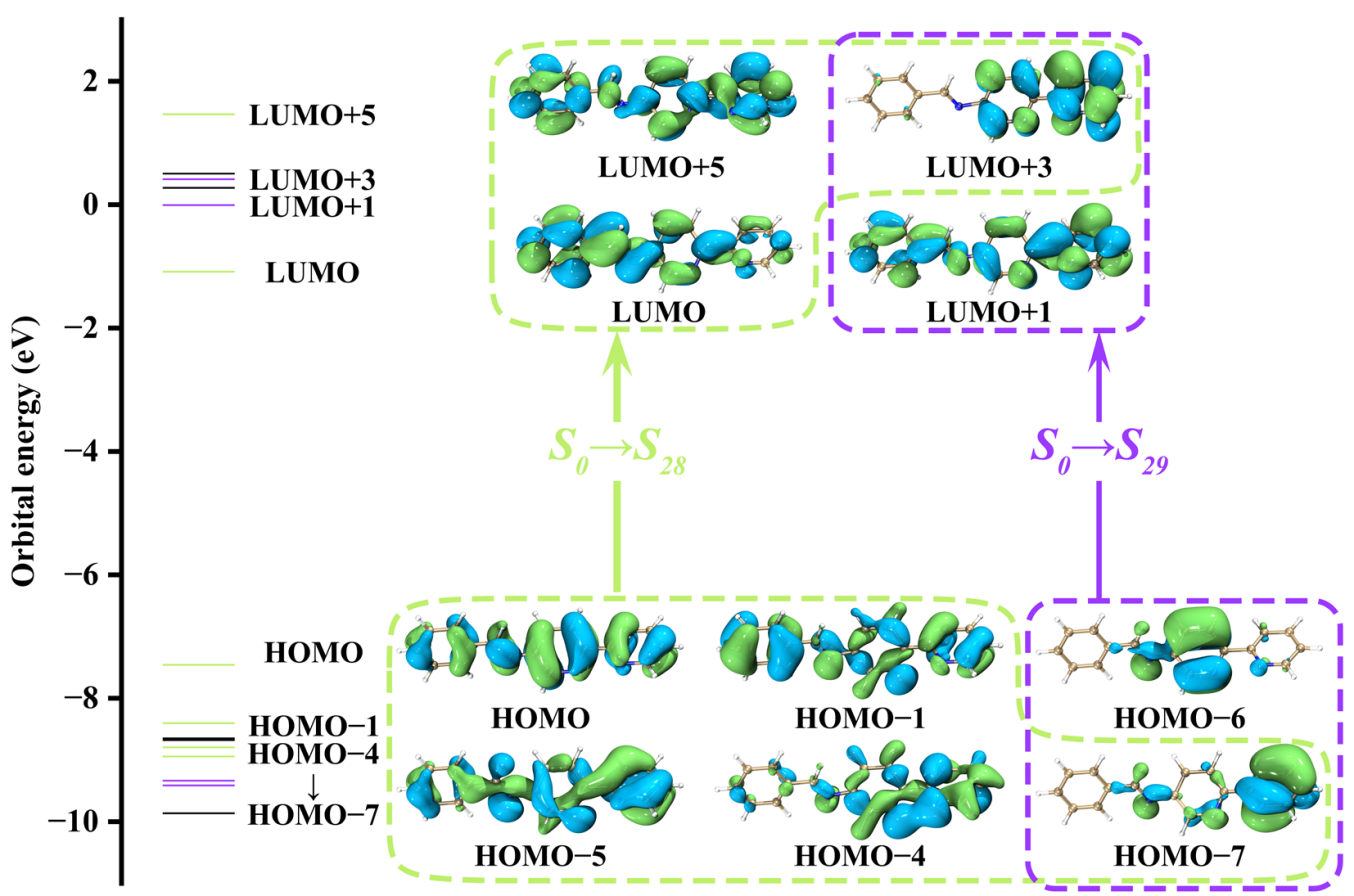
Dominant molecular orbital contributions and energy levels for electronic transitions ($S_{0}\to S_{28}$, and $S_{0}\to S_{29}$) of benzene-C=N-2,2′-Bipyridine(S). The orbital isovalue is set to 0.025 a.u.

Here, let us provide the example of benzene-C=N-2,2′-Bipyridine(S), Table 2 and Figure 2 and Figure 3. For the transition $S_{0}\to S_{1}$, the electron cloud of the participating occupied orbitals (primarily HOMO, HOMO−5, and HOMO−8) is relatively delocalized. Upon excitation, the electron redistributes toward the LUMO, where the electron cloud becomes more localized on the benzene motif. Consequently, a significant electron transfer from the 2,2′-Bipyridine(S) motif to the benzene motif is observed ($\Delta q_{Ph\to UnitB}=-0.1058$). A similar trend occurs for the $S_{0}\to S_{29}$ transition. The initial density, almost entirely localized on the 2,2′-Bipyridine(S) motif, is promoted to the more delocalized LUMO+1 (mainly contributed by the HOMO−6 → LUMO+1 transition, 42.2%), leading to a substantial Δq of −0.0893.

In contrast, the electron transfer behavior is altered or even reversed in other excited states. For $S_{0}\to S_{4}$, the net electron transfer is negligible. This is attributed to the balanced spatial distribution of the initial orbitals (where the 2,2′-Bipyridine(S) motif localized HOMO−6 contributes only 6.3%) and the target orbitals (the LUMO/LUMO+1 pair, which collectively exhibit a relatively uniform electron distribution). Crucially, a reversal of electron transfer is observed for $S_{0}\to S_{20}$ and $S_{0}\to S_{28}$. In the case of $S_{0}\to S_{20}$, although the visual transition scheme is complex, the quantitative charge analysis ($\Delta q_{Ph\to UnitB}=0.0819$) clearly indicates a transfer from the benzene motif to the 2,2′-Bipyridine(S) motif, driven by the localization of LUMO+2 and LUMO+3 on the 2,2′-Bipyridine(S) motif. Similarly, for $S_{0}\to S_{28}$, the participation of orbitals specifically localized on the 2,2′-Bipyridine(S) motif (such as HOMO−4, −5, −7, and LUMO+3) counteracts the primary polarity bias, resulting in a net electron transfer from the benzene motif to the 2,2′-Bipyridine(S) motif.

In this section, the last task is “How to apply these rules into the practical design of solar energy absorption and conversion materials?” In fact, this is very simple and only includes three steps:

Step 1: Point out the wavelength you are interested in. Please pay attention to the fact that the solar spectrum is in a broad range from 300 nm to 2500 nm, which covers a huge width with more than 2000 nm [24]. In this broad range, the electron transfer directions are often different due to different exciting lights. So, the users must point out the wavelength you are interested in.

Step 2: According to this wavelength, check the charge transfer amount and electron transfer direction of various building blocks.

Step 3: Select the building block that can be inserted into your COF system.

In the following sections, the usages are always the same.

### 2.2. Simplest Model K01: hv/e^−^* Behavior Analysis in D-L-A Linear Model

The linear D-L-A model is refined from the simplest photocatalytic D-A system. Compared to the D-A model, the D-L-A model provides more practical guidance for the synthetic design of COFs. Within this cluster-based (fragment-based) model, “D” and “A” in the D-L-A framework denote the structural units, and the “L” refers to the linker, which is the chemical bonds formed during COF synthesis. In the D-L-A model, the designations of “D” and “A” follow the donor–acceptor electronegativity relationship of the two monomers in their ground state when they are directly linked via C-C single bonds. However, in the excited state, the donor–acceptor relationship established in the ground state does not imply that the transfer of excited electrons is invariably from the donor to the acceptor. This transfer direction is closely dependent on the excitation wavelength, and a variation in the excitation wavelength may even cause the excited electrons to transfer in the opposite direction, namely, from the acceptor to the donor. The introduction of the linker complicates the D-A relationship, and the imine linker and the acylhydrazone linker investigated in this work, acting as highly polar linking groups, alter the D-A interaction.

**Figure 4:**
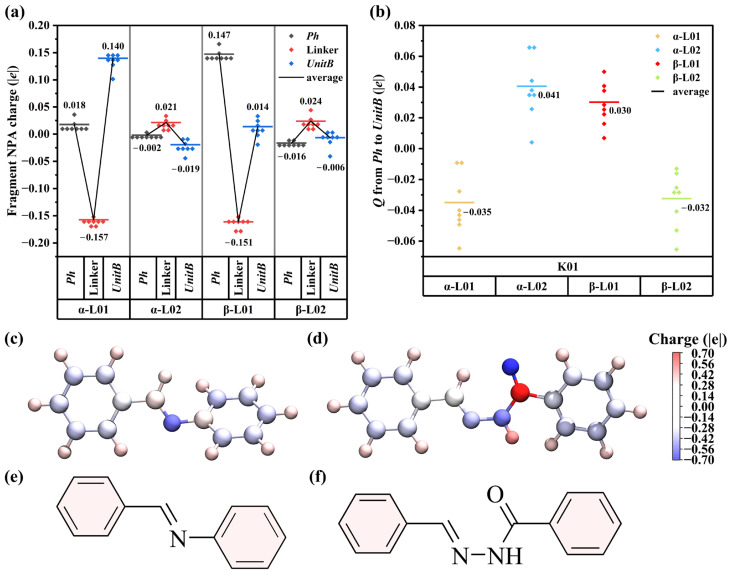
(**a**) The fragment NPA charge in the ground state for K01 models with different linkers (L01, L02, L01, and L02). Diamond symbols represent individual points; horizontal lines indicate average values. (**b**) $Q_{Ph\to UnitB}$ for K01 models. NPA atomic charge distributions visualized by atom coloring for the (**c**) imine linker and (**d**) arylhydrazone linker systems, respectively (using ***Ph*** as an example). Chemical structures of the (**e**) imine linker example and (**f**) acylhydrazone linker example.

In the absence of photon input, the NPA charge distribution in imine-linked systems is predominantly dictated by the polarity and orientation of the imine linker [25]. The D-L-A framework exhibits a “peak–valley–peak” charge distribution pattern, as shown in Figure 4a, where the imine linker serves as the primary site for electron accumulation. Although the relative heights of the surrounding “peaks” differ, this variation depends weakly on the intrinsic donor/acceptor character of the structural units and is instead dominated by the orientation of the imine linker. By reversing the imine direction, the average NPA charge on ***Ph*** obtained for different ***UnitB*** increases from +0.019 |*e*| (nearly neutral) to +0.148 |*e*| (typical positive), while that of ***UnitB*** decreases from +0.139 |*e*| (typical positive) to +0.014 |*e*| (nearly neutral), as shown in Figure 4a. This phenomenon is determined by the dipole moment of the imine linker (directed from N to C, Figure 4c,e) and the N atom serving as the connection site to the adjacent structural unit, which allows for a covalent connection between the N atom and the adjacent unit. Consequently, in the ground state, the N atom not only establishes a significant internal polarity within the linker but also constructs a pronounced polarity at the linker-Unit (L-A or L-D), with the NPA charge difference between the linker and ***UnitB*** reaching as high as ~0.3 |*e*|, as shown in Figure 4a. In contrast, the acylhydrazone-linked system exhibits a mild “valley–peak–valley” charge distribution, as shown in Figure 4a. Although the acylhydrazone bond contains a highly polar C=O group, the presence of carbon atoms at both ends of the linker ensures that the polarity distribution remains largely confined to the linker itself, rather than extending into the structural units as is the case with the imine linker, as shown in Figure 4d,f. Nevertheless, the static charge distribution in acylhydrazone-based D-L-A models remains governed by the linker’s dipole orientation [26,27], consistent with the trends observed in imine-linked systems.

When photoexcitation is introduced into the K01 model, the sign of $Q_{Ph\to UnitB}$ is correlated with the polarity matching of the linker, as shown in Figure 4b. Based on the properties of ***Ph*** and ***UnitB***, the dipole moment is oriented from ***UnitB*** toward ***Ph*** (***Ph*** ← ***UnitB***), designating ***Ph*** as the nominal electron donor. In the α-L01 and β-L02 cases, where the linker polarity aligns with the ***Ph*** ← ***UnitB*** direction, $Q_{Ph\to UnitB}$ is negative, indicating that the optical net electron transfer aligns with the orientation of the dipole moment (***Ph*** ← ***UnitB***). In contrast, for the α-L02 and β-L01 cases, where the linker polarity opposes the ***Ph*** ← ***UnitB*** direction, $Q_{Ph\to UnitB}$ becomes positive, indicating an optical net electron transfer that follows the dipole of the linker (***Ph*** → ***UnitB***). Furthermore, when the linker polarity is matched, the magnitude of $Q_{Ph\to UnitB}$ for imine groups increases from +0.030 |*e*| to +0.035 |*e*|, as shown in Figure 4b, the phenomenon not observed in the acylhydrazone-linked systems.

### 2.3. K02 Model: Chemical Environment Correction

The K02 model extends the K01 framework by increasing the local connectivity of ***Ph*** and ***UnitB*** through the attaching additional linkers. Specifically, ***Ph*** is connected to three linkers and ***UnitB*** to two linkers. This model is used to probe the influence of surrounding linkers on $Q_{Ph\to UnitB}$.

**Figure 5:**
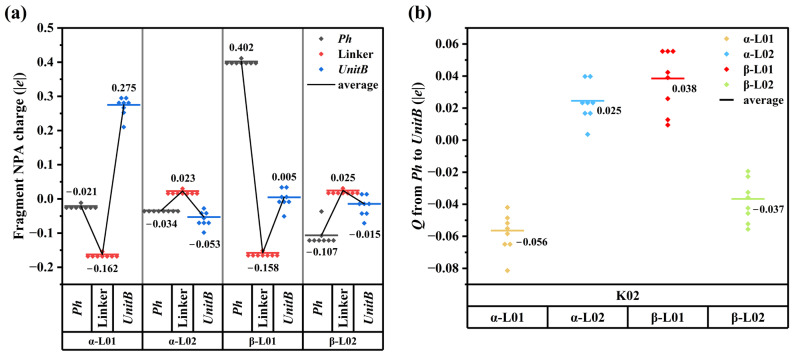
(**a**) The fragment NPA charge in the ground state for K02 models with different linkers (L01, L02, L01, and L02). Diamond symbols represent individual points; horizontal lines indicate average values. (**b**) $Q_{Ph\to UnitB}$ for K02 models.

In the absence of photoexcitation, imine and acylhydrazone linkers exhibit both common and distinct electronic behaviors. In the imine-linked K02 model, the polarization effect of the N atom exhibits a clear additive character. DFT calculations reveal that the direct covalent linkage between the N atom and the adjacent structural unit establishes an effective channel for electron transfer across the linker-Unit connection. For structural units directly bonded to the N atom, the NPA static charge increases approximately linearly with the number of connections. In the α-L01 model, as the number of linkers connected to ***UnitB*** increases from one (K01) to two (K02), the average NPA charge on ***UintB*** increases from +0.139 |*e*| to +0.275 |*e*|, as shown in Figure 5a. In the β-L01 case, where ***Ph*** is connected to three linkers, the average NPA charge on ***Ph*** increases from +0.148 |*e*| (K01) to +0.402 |*e*| in (K02). This increase in charge indicates that multiple polar imine linkers, which connect the structural units via their N termini, cumulatively withdraw electrons from these units, resulting in a highly polarized state of these units.

In stark contrast to the corresponding imine-linked models, the acylhydrazone-linked K02 models exhibit charge polarization largely confined within the linker. Despite the strong polarity of the C=O bond within the acylhydrazone, its polarity remains strictly confined within the linker owing to carbon atoms at both ends, resulting in far smaller perturbations to the ground-state charge distribution on adjacent fragments compared to the imine-linked case. The average NPA charge on ***Ph*** is more negative in β-L02 (−0.107 |*e*|) than in α-L02 (−0.034 |*e*|), consistent with the greater electronegativity of the β-L02 end group (***Ph***-C(NH)=O) compared to the α-L02 terminus (***Ph***-C=N). Similarly, that on ***UnitB*** changes from −0.015 |*e*| (C=N-***UnitB***) to −0.053 |*e*| (O=(H-N)C-***UnitB***), as shown in Figure 5a.

The progression from K01 to K02 reveals a key design principle for COF nodes: the imine bond enables a tunable charge-modulation mechanism that propagates across the linker via the strong polarity of the N atom, whereas the acylhydrazone bond confines charge polarization predominantly within the linker itself.

Under photoexcitation, $Q_{Ph\to UnitB}$ continues to follow the polarity-directed orientation established in the K01 D-L-A model. In the imine-linked systems, the absolute value of $Q_{Ph\to UnitB}$ increases modestly relative to K01: from −0.035 |*e*| to −0.056 |*e*| for α-L01, and from +0.030 |*e*| to +0.038 |*e*| for β-L01, as shown in Figure 5b. In contrast, the acylhydrazone-linked systems exhibit more substantial variations. For α-L02, where the linker polarity opposes the inherent polarity direction from ***Ph*** to ***UnitB***, $Q_{Ph\to UnitB}$ decreases sharply from +0.41 |*e*| to +0.025 |*e*|, Figure 5b. For β-L02, the absolute value of $Q_{Ph\to UnitB}$ rises from −0.032 |*e*| to −0.037 |*e*|, as shown in Figure 5b. These trends indicate that, in the acylhydrazone system, $Q_{Ph\to UnitB}$ is amplified when the linker polarity aligns with the inherent polarity direction from ***Ph*** to ***UnitB***, while misalignment leads to pronounced suppression.

### 2.4. Model K03: Symmetric Central Node Model

The K03 model utilizes **Ph** as the central unit and constructs a highly symmetric molecular fragment by attaching three **UnitB** and three linkers. This model is designed to mimic the local environment of the three connected nodes in the COF lattice [28].

In the imine-linked systems, completing the surrounding linker–***UnitB*** environment leads to changes in the average NPA charge on ***Ph*** depending on the linker orientation. For α-L01, the charge on ***Ph*** shifts from −0.021 |*e*| to +0.001 |*e*| (becoming less negative), while for β-L01, it increases from +0.402 |*e*| to +0.421 |*e*| (becoming more positive). These trends indicate that a more complete local coordination enhances the overall electron-withdrawing effect of the linkers on the central ***Ph*** node. Compared to K01, charge extraction from ***UnitB*** in K03 is also amplified.

**Figure 6:**
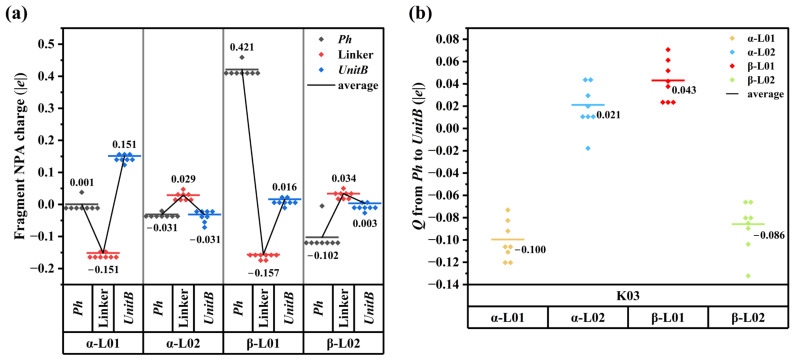
(**a**) The fragment NPA charge in the ground state for K03 models with different linkers (L01, L02, L01, and L02). Diamond symbols represent individual points; horizontal lines indicate average values. (**b**) $Q_{Ph\to UnitB}$ for K03 models.

In the acylhydrazone-linked system, the average charge distribution in K03 remains highly similar to that in K02. The average NPA charge on ***Ph*** shows small positive shifts of +0.003 |*e*| for α-L02 and +0.005 |*e*| for β-L02, as shown in Figure 6a. The variations on ***UnitB*** are slightly larger, amounting to +0.022 |*e*| for α-L02 and +0.018 |*e*| for β-L02, as shown in Figure 6a. These modest shifts further confirm the strong confinement of charge polarization within the acylhydrazone linker, which effectively prevents significant changes in the charge distribution of the central node due to surrounding linkers.

Under photoexcitation, $Q_{Ph\to UnitB}$ continues to follow the polarity-directed pathway established in the K01 D-L-A model. However, in K03, the two cases in which the linker polarity aligns with the intrinsic ***Ph***–***UnitB*** polarity (α-L01 and β-L02) exhibit nearly doubles in the absolute value of $Q_{Ph\to UnitB}$ compared with K02: from −0.056 |*e*| to −0.100 |*e*| for α-L01, and from −0.037 |*e*| to −0.086 |*e*| for β-L02, as shown on Figure 6b. In contrast, the polarity-misaligned cases show minimal change in the absolute value of $Q_{Ph\to UnitB}$. These results reveal a key distinction in the symmetric K03 environment. Compared to the K01 D-L-A model in K01, the presence of three equivalent ***UnitB*** donors in K03 leads to a nearly linear enhancement in $Q_{Ph\to UnitB}$ when ***Ph*** acts as the acceptor in the polarity-matched α-L01 and β-L02 cases. Conversely, when ***Ph*** acts as the donor (α-L02; β-L01), $Q_{Ph\to UnitB}$ shows minimal change relative to K01 and K02. The values are +0.021 |*e*| (α-L02) and +0.043 |*e*| (β-L01), which do not significantly exceed the K01 (+0.030 |*e*|) or K02 (+0.038 |*e*|) values, and remain much smaller than the corresponding acceptor case in β-L02 (−0.086 |*e*|), as shown in Figure 6b.

### 2.5. Asymmetric Peripheral Model

In the K04 model, ***Ph*** is positioned at the periphery and connected to only one linker, while ***UnitB*** is placed at the center and connected to two linkers and two additional ***UnitB*** units. This model creates an asymmetric environment that isolates the peripheral node while partially saturating the central one.

In the absence of photoexcitation, the imine-linked system shows that the average NPA charge distribution on the peripheral ***Ph*** deviates only modestly from that observed in K01, with the electron-withdrawing behavior of the linker remaining largely unchanged compared to K01. In contrast, the average charge distribution of the central ***UnitB***, which attaches two linkers, is closer to that observed in K02. For the acylhydrazone-linked system, the average NPA charge on the peripheral ***Ph*** likewise exhibits only minor differences relative to K01, and the linker’s NPA charge distribution follows a similar pattern.

**Figure 7:**
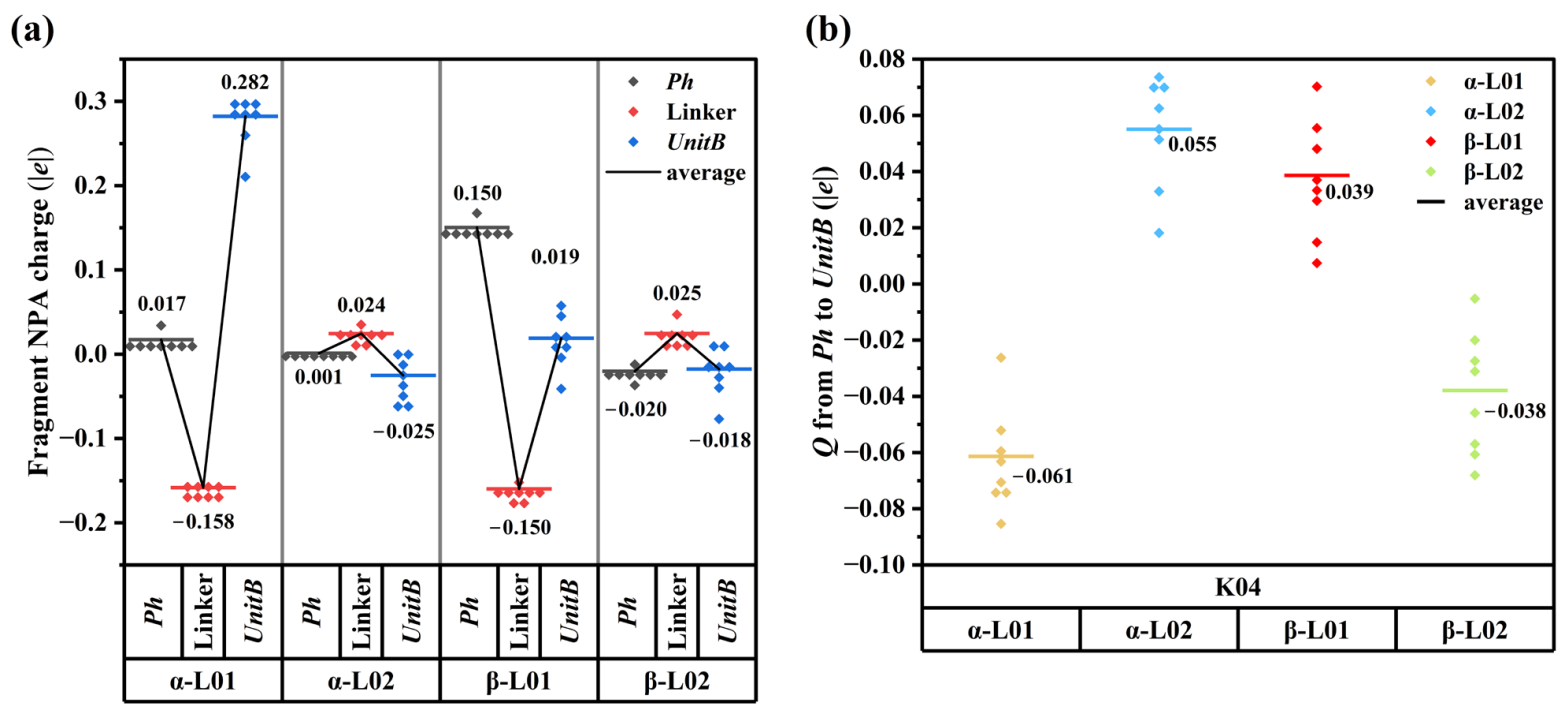
(**a**) The fragment NPA charge in the ground state for K04 models with different linkers (L01, L02, L01, and L02). Diamond symbols represent individual points; horizontal lines indicate average values. (**b**) $Q_{Ph\to UnitB}$ for K04 models.

Under photoexcitation, the K04 models reveal partly different trends compared to K03. Here, a single donor (***UnitB*** at the center) transfers electrons to two peripheral acceptors ***Ph***. In the imine-linked system, when the linker polarity aligns with the D → A polarity direction, the absolute of $Q_{Ph\to UnitB}$ nearly doubles relative to K01 (from −0.035 |*e*| in K01 to −0.061 |*e*| in K04, Figure 4b and Figure 7b, consistent with the doubled number of acceptor sites. Conversely, when the polarity of the linker directs A → D, the absolute of $Q_{Ph\to UnitB}$ shows only modest change relative to the linear K01 model (from 0.030 |*e*| in K01 to 0.039 |*e*| in K04), less than the near-linear enhancement observed in the D → A direction.

In the acylhydrazone-linked system, $Q_{Ph\to UnitB}$ remains broadly similar to that observed in the K01 D-L-A model, showing no significant enhancement or suppression. This contrast highlights that the acylhydrazone linker strongly confines charge polarization within the linker, limiting its influence on adjacent units even in branched models, whereas the imine linkage enables significant enhancement in the absolute of $Q_{Ph\to UnitB}$ in multi-acceptor geometries. The applicability of these rules across the four fragment models is summarized in Table 3.

Two key rules emerge from these observations:

**Rule 1**: The direction of $Q_{Ph\to UnitB}$ is highly consistent with the polarity direction of the linker across all models.

**Rule 2**: When the dipole moment of the linker aligns with the intrinsic dipole moment direction between ***Ph*** and ***UnitB***, the absolute value of $Q_{Ph\to UnitB}$ is significantly enhanced.

The applicability of these rules across the four fragment models is summarized in Table 3.

Notably, for the acylhydrazone linker in K01 and K02 models, the enhancement effect predicted by Rule 2 is partially inhibited. This is primarily attributed to the strong electronegativity of the carbonyl oxygen (C=O), which induces significant charge localization within the linker moiety. This electronic isolation prevents the linker’s dipole from effectively coupling with the broader donor–acceptor backbone. Furthermore, the spatial orientation of the C=O groups relative to the framework’s intrinsic polarity plays a decisive role. In K01 and K04, the geometric arrangement leads to a misalignment (or partial cancellation) between the linker’s local dipole and the unit’s dipole, thereby inhibiting the synergistic enhancement. In contrast, the configurations of K02 and K03 allow for a more favorable spatial alignment, which preserves the additive effect of the dipoles to a greater extent, even in the presence of charge localization.

Furthermore, considering that practical photocatalysis often occurs in solution, the potential influence of the solvent’s dielectric constant on linker polarity warrants discussion. While a solvent environment can modulate the magnitude of dipole moments through electronic polarization, the fundamental direction of electron transfer is primarily dictated by the intrinsic chemical nature of the linker, as established by Rule 1 and Rule 2. According to the Franck–Condon principle, vertical excitation occurs so rapidly that solvent reorganization primarily affects the stabilization of the excited state rather than reversing the initial transition density flow. This is consistent with previous studies on similar COF systems [29,30], which demonstrate that gas-phase trends in charge transfer remain qualitatively robust in condensed phases.

## 3. Methods

### 3.1. Donor–Linker–Acceptor Electron Transfer Models

In this study, we developed a fragment-based deconstruction computational model to simulate and analyze electron transfer in COF skeletons [11]. Recognizing that both ground-state geometry optimizations and excited-state calculations for full COF lattices are computationally prohibitive due to their long-range order and high atom counts [31,32], we simplified these complex structures into specific donor–linker–acceptor (D-L-A) pairs.

To ensure the influence of surrounding structures on the electronic architecture and excited-state electron transfer of these D-L-A pairs, we designed four multi-level fragment models, K01–K04:

(1) K01 (*simplified core unit*): Composed of two body fragments coupled by a single linker, which is named the donor–linker–acceptor (D-L-A) model, serving as the minimum physical unit to define charge transition properties.
(2) K02 (*chemical environment correction model*): Built upon K01 by adding adjacent linking groups to examine the influence of surrounding linkers on the D-L-A electron transfer.
(3) K03 (*symmetric central node model*): Centered on a trifunctional node extending outward, containing four body units and three linkers.
(4) K04 (*asymmetric peripheral model*): Places the trifunctional central node at the edge of the fragment, containing three body units and two linkers.

In the present work, phenyl unit (***Ph***) was employed as the trifunctional nodes, combined with eight types of bifunctional linkers as shown in Scheme 1. By conducting a systematic study of 216 topological combinations, we mapped the electron flow pathways and investigated the relationship between ground-state pre-polarization and the direction of excited-state electron transfer.

### 3.2. Computational Details

All the density functional theory (DFT) calculations were performed using the Gaussian 16 quantum chemistry software (Revision A.03) at the level of M06-2X-D3(0)/6-311g(2d,p) [33,34,35,36,37]. The ground-state geometries were fully optimized without symmetry constraints. Subsequently, the excited-state properties were investigated via linear response time-dependent DFT (TD-DFT) at the same level of theory [38]. This procedure follows the vertical excitation approximation, where the first 50 singlet excited states were calculated based on the optimized ground-state ($S_{0}$) geometries to describe the instantaneous electronic transitions. To improve the precision of the excited-state electronic description, the *IOp(9/40 = 4)* keyword was also utilized.

For each excited state, the net electron transfer from ***Ph*** to ***UnitB*** was quantified using the interfragment charge transfer method (IFCT) in the Multiwfn program (version 3.8(dev), update date: 14 August 2025) [39,40], which evaluates the net electron transfer from one fragment to another based on the difference in electron density between the ground and excited states from a probabilistic perspective.

The effective optical net electron transfer from ***Ph*** to ***UnitB*** ($Q_{Ph\to UnitB}$) is the average of the net electron transfers (Δq) from IFCT analysis, weighted by oscillator strength, over the lowest 50 excited states:

(1) $$Q_{Ph\to UnitB}=\frac{\sum_{i=1}^{50}\Delta q_{i,Ph\to UnitB}\times f_{i}}{\sum_{i=1}^{50}f_{i}}$$

where $f_{i}$ is the oscillator strength for the transition to the *i*-th excited state from the ground state, and $\Delta q_{i,Ph\to UnitB}$ is the net electron transfer from ***Ph*** to ***UnitB*** in the *i*-th excited state (positive values indicate net from ***Ph*** to ***UnitB***). This weighted quantity reflects the contribution of each transition to the overall optical net charge separation process, with stronger transitions (higher $f_{i}$) having greater influence.

## 4. Conclusions

In summary, this study systematically reveals that linker polarity is a decisive factor in regulating the effective optical net electron transfer ($Q_{Ph\to UnitB}$) in COF fragment models. By deconstructing the complex COF skeletons into D-L-A models, we established that the polarity orientation of imine and acylhydrazone linkers can effectively modulate the direction and magnitude of charge transfer. Based on our findings, several key physical recommendations for the rational design of photocatalytic COFs are proposed:

Polarity alignment principle: To maximize charge separation efficiency, the intrinsic dipole moment of the linker should be strategically aligned with the desired D → A polarization vector. In imine-linked systems, such alignment significantly enhances electron transfer magnitude, whereas a mismatch leads to suppressed or negligible electron transfer.

Linkage-specific selection: Imine linkages are more sensitive to geometric symmetry and polarity matching, making them ideal for systems requiring fine-tuned transition dipole moments. In contrast, acylhydrazone linkages exhibit stronger charge localization, which may be more suitable for stabilizing specific radical intermediates in multi-step catalytic processes.

Excitation wavelength awareness: The “D/A” role is not static; it can be reversed in higher-energy excited states due to differing molecular orbital contributions. Therefore, the design must ensure that the “constructive” polarity-matching rule aligns with the peak intensity of the target light source (e.g., the solar spectrum).

Regarding the generality of these conclusions, the polarity-matching framework developed here using D-L-A fragment models provides a fundamental physical basis that is expected to be applicable to broader 2D and 3D COFs. While periodic boundary conditions and long-range packing in crystals may introduce additional delocalization effects, the local electronic environment, which is governed by the linker’s dipole, remains the primary driver of the initial exciton dissociation. Consequently, these rules can serve as a robust screening table for selecting building blocks in high-performance photocatalysts. Future work will focus on integrating these molecular-level rules into multiscale simulations to further bridge the gap between fragment models and macroscale device performance.

## Figures and Tables

**Table 1:** Calculated dipole moments (Debye) for ***Ph***-***UnitB*** systems and their projections along the ***Ph*** → ***UnitB*** direction.

*Ph*	*UnitB*	Dipole Moment (Debye)	Projection ^1^ (Debye)
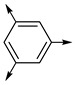	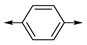	0	0
	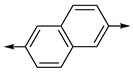	0.02	0.01
	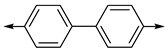	0.00	0.00
	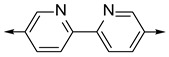	3.05	−0.08
	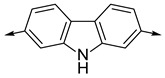	1.55	−0.24
	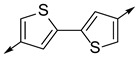	0.30	−0.19
	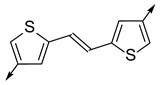	0.24	−0.24
	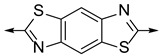	1.26	−1.22

^1^ The projection is taken along the vector pointing from the carbon atom of ***Ph*** directly connected to ***UnitB*** to the carbon atom of ***UnitB*** directly connected to ***Ph***. A positive projection implies the direction from ***Ph*** to ***UnitB***, while a negative projection implies the opposite.

**Table 2:** Calculated absorption wavelengths (λ), oscillator strengths (*f_osc_*), light-harvesting efficiency (LHE), net electron transfer from ***Ph*** to ***UnitB*** ($\Delta q_{Ph\to UnitB}$) and main configurations (with contributions) for the five singlet excited states with the highest oscillator strengths, selected from the first 50 excited states of 2,2′-Bipyridine(S)-N=C-benzene.

Transition	λ (nm)	*f_osc_*	LHE	$\Delta q_{Ph\to UnitB}$	Main Configuration ^1^ (Contribution %)
*S*_0_ → *S*_4_	257.7	0.657	0.940	−0.0005	H−5 → L (20.2%)
					H → L (17.7%)
					H−1 → L+1 (10.1%)
					H−8 → L (10.1%)
					H → L+1 (6.7%)
					H−6 → L (6.3%)
*S*_0_ → *S*_20_	180.0	0.509	0.617	0.0819	H−3 → L+4 (23.7%)
					H−2 → L+2 (9.1%)
					H−1 → L+2 (8.5%)
					H−2 → L+3 (7.7%)
					H−4 → L+2 (5.7%)
					H−2 → L+4 (5.1%)
*S*_0_ → *S*_1_	306.5	0.483	0.615	−0.1058	H → L (65.4%)
					H−5 → L (9.8%)
					H−8 → L (5.7%)
*S*_0_ → *S*_28_	172.6	0.181	0.397	0.0130	H−5 → L+3 (13.2%)
					H → L+5 (12.6%)
					H−1 → L+3 (9.4%)
					H−4 → L+3 ( 8.9%)
					H−7 → L (5.2%)
*S*_0_ → *S*_29_	171.1	0.149	0.371	−0.0893	H−6 → L+1 (42.2%)
					H−7 → L+3 (9.9%)

^1^ H and L represent the highest occupied molecular orbital (HOMO) and the lowest unoccupied molecular orbital (LUMO), respectively.

**Table 3:** Applicability of Rule 1 and Rule 2 in the four fragment models under imine and acylhydrazone linkers. √ = rule satisfied; × = rule not satisfied.

Model	Rule 1		Rule 2	
	Imine	Acylhydrazone	Imine	Acylhydrazone
K01	√	√	√	×
K02	√	√	√	√
K03	√	√	√	√
K04	√	√	√	×

## Data Availability

The computational data supporting the findings is available from the corresponding author upon reasonable request.

## Supplementary Materials

The following supporting information can be downloaded at https://www.mdpi.com/article/10.3390/molecules31071179/s1, Table S1: Calculated net electron transfer ($\Delta q_{Ph\to UnitB}$) for the five excited states with the highest Light Harvesting Efficiency (LHE) among the first 50 states of model K01, along with the effective optical net electron transfer from ***Ph*** to ***UnitB*** ($Q_{Ph\to UnitB}$) for the first 50 excited states; Table S2: Calculated net electron transfer ($\Delta q_{Ph\to UnitB}$) for the five excited states with the highest Light Harvesting Efficiency (LHE) among the first 50 states of model K02, along with the effective optical net electron transfer from ***Ph*** to ***UnitB*** ($Q_{Ph\to UnitB}$) for the first 50 excited states; Table S3: Calculated net electron transfer ($\Delta q_{Ph\to UnitB}$) for the five excited states with the highest Light Harvesting Efficiency (LHE) among the first 50 states of model K03, along with the effective optical net electron transfer from ***Ph*** to ***UnitB*** ($Q_{Ph\to UnitB}$) for the first 50 excited states; Table S4: Calculated net electron transfer ($\Delta q_{Ph\to UnitB}$) for the five excited states with the highest Light Harvesting Efficiency (LHE) among the first 50 states of model K04, along with the effective optical net electron transfer from ***Ph*** to ***UnitB*** ($Q_{Ph\to UnitB}$) for the first 50 excited states; OptimizedGeometries: Optimized Cartesian coordinates of all calculated structures in XYZ format.
