# Supplementary material for: Computational Study of Linker Polarity Effects on Optical Electron Transfer in Imine- and Acylhydrazone-Linked Covalent Organic Frameworks Using Fragment Models
Source: Molecules. 2026 Apr 2;31(7):1179. doi: 10.3390/molecules31071179 (PMC13074386; doi:10.3390/molecules31071179)
Supplement: Supplementary file 1 [file molecules-31-01179-s001.zip › Supporting Information.pdf]

Supporting Information

Computational Study of Linker Polarity Effects on Optical Electron Transfer in Imine- and Acylhydrazone-Linked Covalent Organic Frameworks Using Fragment Models

Junjin Chen, Dongdong Qi and Jianzhuang Jiang.

Contents

Table S1. Calculated net electron transfer ($\Delta q_{\boldsymbol{Ph \rightarrow UnitB}}$) for the five excited states with the highest Light Harvesting Efficiency (LHE) among the first 50 states of model K01, along with the effective optical net electron transfer from ***Ph*** to ***UnitB*** ($Q_{\boldsymbol{Ph \rightarrow UnitB}}$) for the first 50 excited states. (Pages S2-S10)

Table S2. Calculated net electron transfer ($\Delta q_{\boldsymbol{Ph \rightarrow UnitB}}$) for the five excited states with the highest Light Harvesting Efficiency (LHE) among the first 50 states of model K02, along with the effective optical net electron transfer from ***Ph*** to ***UnitB*** ($Q_{\boldsymbol{Ph \rightarrow UnitB}}$) for the first 50 excited states. (Pages S11-S19)

Table S3. Calculated net electron transfer ($\Delta q_{\boldsymbol{Ph \rightarrow UnitB}}$) for the five excited states with the highest Light Harvesting Efficiency (LHE) among the first 50 states of model K03, along with the effective optical net electron transfer from ***Ph*** to ***UnitB*** ($Q_{\boldsymbol{Ph \rightarrow UnitB}}$) for the first 50 excited statesX. (Pages S20-S28)

Table S4. Calculated net electron transfer ($\Delta q_{\boldsymbol{Ph \rightarrow UnitB}}$) for the five excited states with the highest Light Harvesting Efficiency (LHE) among the first 50 states of model K04, along with the effective optical net electron transfer from ***Ph*** to ***UnitB*** ($Q_{\boldsymbol{Ph \rightarrow UnitB}}$) for the first 50 excited states. (Pages S29-S36)

Table S1. Calculated net electron transfer ($\Delta q_{Ph\rightarrow UnitB}$) for the five excited states with the highest Light Harvesting Efficiency (LHE) among the first 50 states of model K01, along with the effective optical net electron transfer from ***Ph*** to ***UnitB*** ($Q_{Ph\rightarrow UnitB}$) for the first 50 excited states.

hv (nm)	LHE	***UnitB***[1]	Linker	***Ph***	$\Delta q_{Ph\rightarrow UnitB}$ / $Q_{Ph\rightarrow UnitB}$ (|*e*|)
248.4	0.776	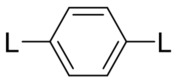	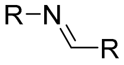	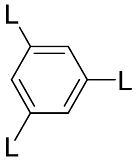	−0.0050
182.5	0.693				0.0189
196.6	0.336				−0.0904
300.8	0.335				−0.0977
176.6	0.275				0.0516
Weighted					−0.0069
248.4	0.776	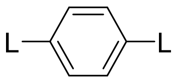	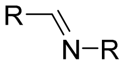	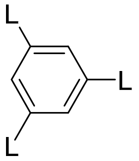	0.0050
182.5	0.693				−0.0189
196.6	0.336				0.0904
300.8	0.335				0.0977
176.6	0.275				−0.0516
Weighted					0.0068
265.0	0.776	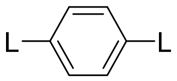	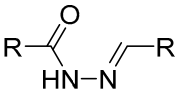	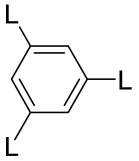	0.0507
178.0	0.693				0.0749
181.6	0.336				−0.0477
196.5	0.335				0.2008
168.9	0.275				−0.1975
Weighted					0.0378
265.0	0.852	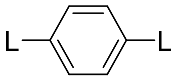	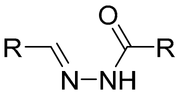	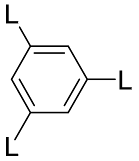	−0.0507
178.0	0.539				−0.0749
181.6	0.350				0.0477
196.5	0.302				−0.2008
168.9	0.299				0.1975
Weighted					−0.0378
251.2	0.852	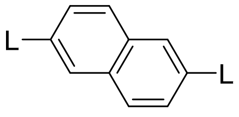	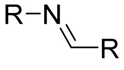	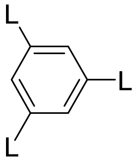	−0.0023
205.0	0.539				−0.0323
312.4	0.350				−0.1295
264.2	0.302	(S)			−0.0463
183.0	0.299				−0.2152
Weighted					−0.0345
246.9	0.862	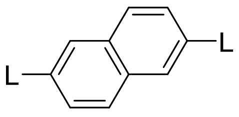	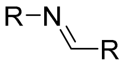	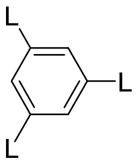	0.0158
204.9	0.856				−0.0207
309.8	0.550				−0.1332
197.8	0.301	(Z)			−0.2762

170.9	0.296				0.1585
Weighted					−0.0204
242.0	0.875	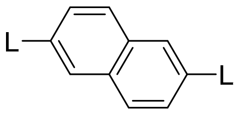 (S)	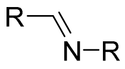	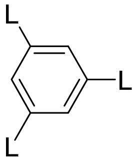	0.0376
212.3	0.826				0.0586
303.6	0.504				0.1121
267.4	0.335				−0.0021
191.5	0.268				0.0084
Weighted					0.0453
242.4	0.869	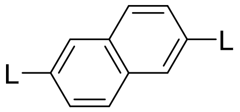 (Z)	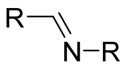	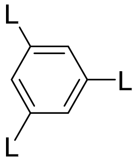	0.0526
192.0	0.563				0.0903
310.9	0.543				0.1176
175.9	0.462				0.1043
176.9	0.379				0.2626
Weighted					0.0594
213.5	0.898	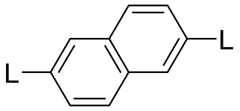 (S)	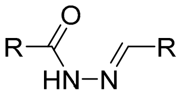	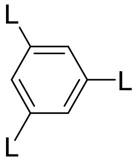	−0.0583
269.3	0.523				0.0490
258.0	0.520				0.0057
196.6	0.406				−0.0486
179.0	0.321				0.0375
Weighted					0.0226
270.3	0.807	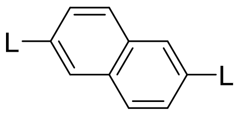 (Z)	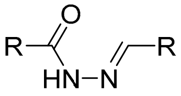	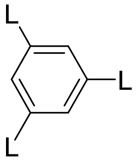	0.0546
214.7	0.705				−0.0517
194.6	0.466				−0.0600
260.8	0.464				0.0120
198.6	0.362				0.0414
Weighted					0.0327
251.5	0.815	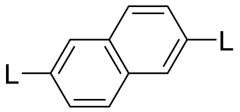 (S)	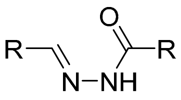	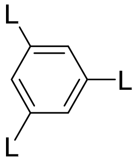	−0.0828
295.8	0.746				−0.0338
170.9	0.501				0.2387
194.8	0.436				−0.0396
173.0	0.384				0.1660
Weighted					−0.0235
213.0	0.841	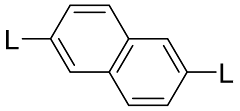 (Z)	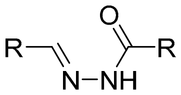	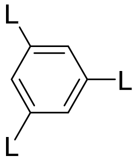	−0.1206
250.1	0.712				−0.0578
285.3	0.475				−0.0420
274.9	0.473				−0.0271
168.6	0.468				0.1251
Weighted					−0.0266
258.1	0.763	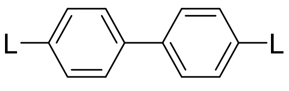	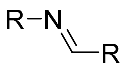	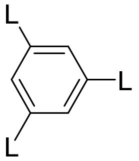	−0.0123
182.8	0.743				−0.0089
310.2	0.669				−0.1190
185.1	0.462				0.0273

198.9	0.425				−0.2012
Weighted					−0.0417
268.9	0.864	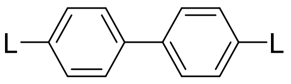	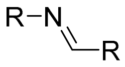	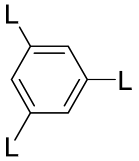	−0.0287
183.4	0.802				0.0443
181.8	0.651				0.0043
306.0	0.536				0.1144
160.5	0.398				−0.0048
Weighted					0.0251
269.8	0.873	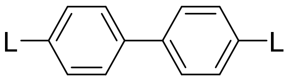	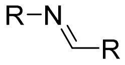	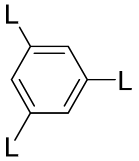	0.0446
182.7	0.775				0.0152
181.7	0.663				0.0452
280.4	0.628				−0.0002
197.2	0.252				0.1398
Weighted					0.0377
285.0	0.911	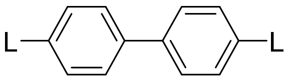	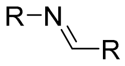	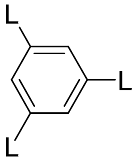	−0.0380
182.9	0.806				0.0426
182.0	0.592				−0.0045
208.1	0.493				0.0234
169.1	0.350				0.2773
Weighted					−0.0279
257.7	0.940	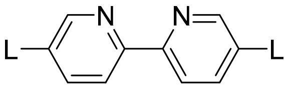 (S)	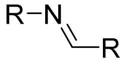	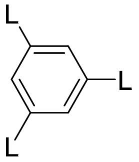	−0.0005
180.0	0.617				0.0819
306.5	0.615				−0.1058
172.6	0.397				0.0130
171.1	0.371				−0.0893
Weighted					−0.0042
271.2	0.779	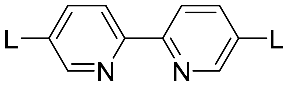 (Z)	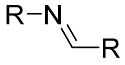	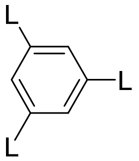	−0.0099
182.4	0.690				0.0009
307.7	0.671				−0.1057
258.2	0.340				−0.0088
172.7	0.290				−0.0299
Weighted					−0.0138
268.0	0.639	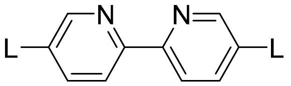 (S)	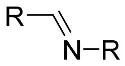	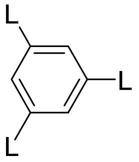	−0.0095
309.8	0.580				0.1297
175.5	0.544				−0.0180
265.6	0.534				−0.0155
173.3	0.377				−0.1652
Weighted					0.0357
173.3	0.737	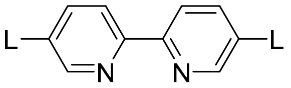 (Z)	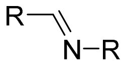	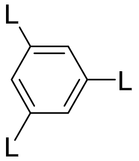	0.0060
277.4	0.581				0.0145
263.5	0.569				−0.0181
313.1	0.419				0.1094

173.9	0.417				0.1643
Weighted					0.0496
272.7	0.625	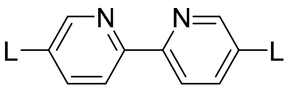	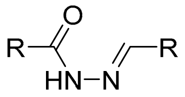	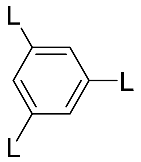	0.0692
171.4	0.612				0.0074
180.0	0.609				0.1367
169.4	0.480	(S)			0.0070
198.5	0.461				0.1797
Weighted					0.0646
274.1	0.919	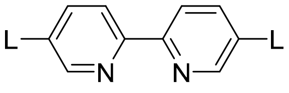	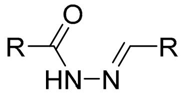	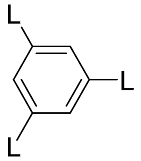	0.0751
172.6	0.594				0.0465
180.0	0.323				0.0994
171.7	0.319	(Z)			0.0812
199.0	0.300				0.1893
Weighted					0.0722
284.5	0.924	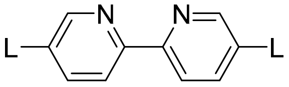	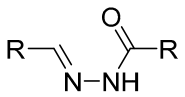	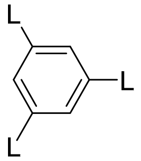	−0.0204
173.6	0.395				0.1043
169.7	0.353				−0.0418
171.3	0.320	(S)			0.1194
278.3	0.293				−0.0127
Weighted					0.0014
280.4	0.919	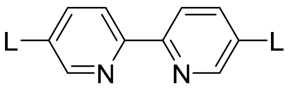	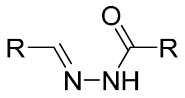	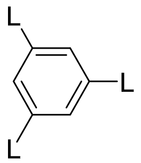	−0.0318
173.6	0.539				0.1171
172.2	0.503				−0.0149
287.1	0.494	(Z)			−0.0117
177.0	0.454				−0.0444
Weighted					−0.0238
317.7	0.934	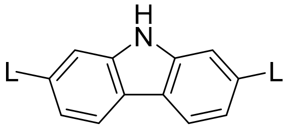	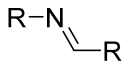	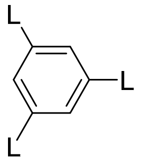	−0.1404
266.5	0.643				−0.0341
209.6	0.511				−0.0769
226.4	0.397				0.0039
178.7	0.244	(S)			−0.0156
Weighted					−0.0615
319.6	0.721	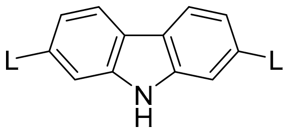	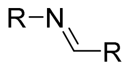	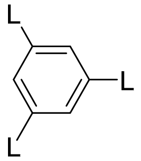	−0.1421
263.9	0.669				−0.0278
178.1	0.611				0.0184
209.3	0.589				−0.1045
227.5	0.565	(Z)			−0.0189
Weighted					−0.0678
306.6	0.710	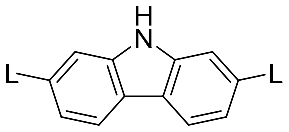	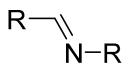	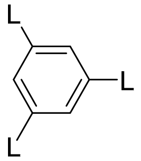	0.0993
279.4	0.710				−0.0213
175.8	0.649				−0.0490
215.1	0.538				0.1247

226.7	0.536	(S)			0.0516
Weighted					0.0278
310.4	0.770	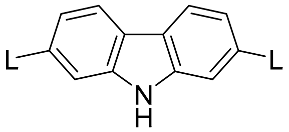	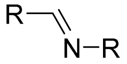	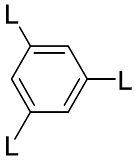	0.0654
281.8	0.685				−0.0097
186.9	0.635				−0.0273
228.6	0.506				0.0508
212.0	0.485	(Z)			−0.0522
Weighted					0.0072
282.0	0.701	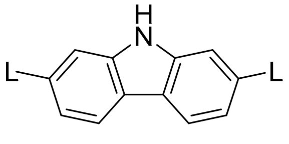	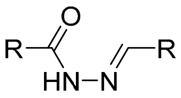	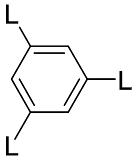	−0.0007
218.2	0.619				−0.0588
273.3	0.552				0.0024
178.2	0.531				0.0771
220.6	0.484	(S)			0.1589
Weighted					0.0200
282.6	0.850	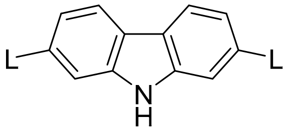	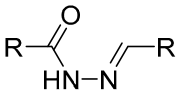	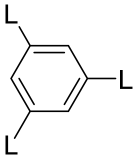	−0.0043
272.5	0.738				0.0053
217.6	0.712				−0.1799
189.7	0.549				−0.0833
215.7	0.463	(Z)			−0.1128
Weighted					−0.0119
294.1	0.791	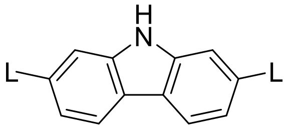	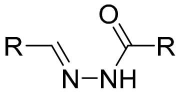	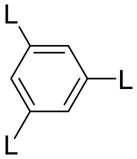	−0.0479
219.5	0.776				−0.1807
226.5	0.660				−0.1204
181.8	0.455				0.0315
178.1	0.426	(S)			−0.0762
Weighted					−0.0599
298.1	0.934	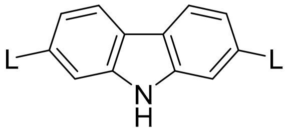	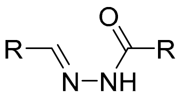	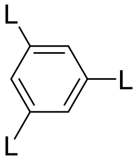	−0.0343
222.4	0.667				−0.1499
187.5	0.559				−0.0218
303.6	0.338				−0.0227
172.0	0.331	(Z)			0.1597
Weighted					−0.0448
352.4	0.907	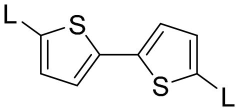	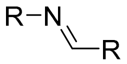	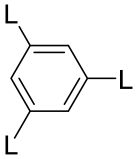	−0.0764
181.9	0.567				0.0443
167.9	0.466				0.2090
173.1	0.385	(S)			−0.0020
210.3	0.346				−0.0412
Weighted					−0.0402
351.4	0.902	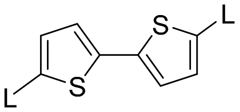	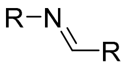	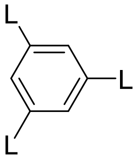	−0.1404
178.3	0.393				0.0037
279.7	0.308				−0.0480
238.0	0.296	(Z)			0.0227

176.6	0.196				0.0118
Weighted					−0.0567
330.9	0.838	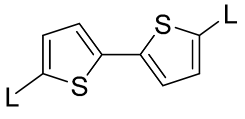 (S)	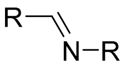	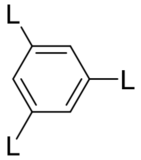	0.0581
175.0	0.431				0.1192
173.6	0.369				−0.0545
160.2	0.263				0.1920
176.3	0.241				0.0155
Weighted					0.0385
333.3	0.887	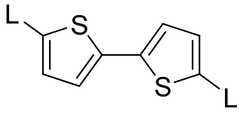 (Z)	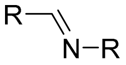	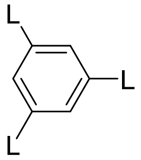	0.0542
175.8	0.400				0.0391
231.0	0.344				0.0495
175.1	0.269				0.1255
202.9	0.242				0.0031
Weighted					0.0389
306.0	0.876	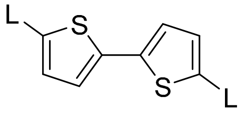 (S)	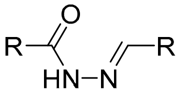	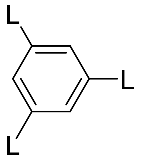	0.0127
265.8	0.589				0.0927
179.5	0.311				0.1539
187.0	0.262				0.3140
200.5	0.229				0.2494
Weighted					0.0515
307.5	0.905	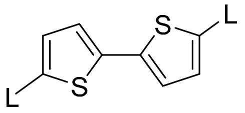 (Z)	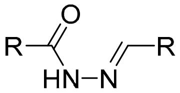	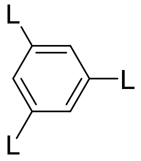	0.0059
179.7	0.352				0.1123
264.6	0.346				0.0865
200.1	0.324				0.2020
185.6	0.257				0.2764
Weighted					0.0388
326.0	0.918	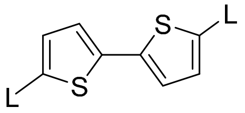 (S)	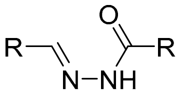	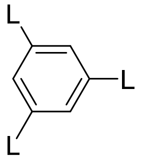	−0.0305
173.2	0.348				0.1568
171.8	0.327				−0.2060
171.3	0.268				0.0456
208.1	0.262				0.0593
Weighted					−0.0191
328.7	0.918	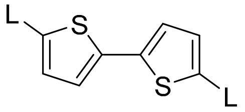 (Z)	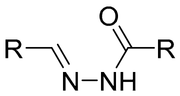	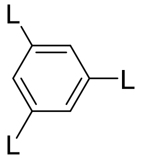	−0.0254
173.9	0.527				0.1431
172.1	0.327				0.1800
208.1	0.279				0.0297
166.3	0.269				−0.1312
Weighted					−0.0088
383.4	0.903	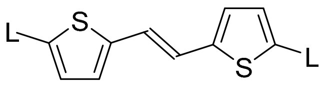 (S)	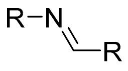	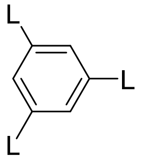	−0.0627
177.7	0.577				0.0183
170.8	0.385				0.1222
215.8	0.233				−0.3090

226.3	0.230				0.0325
Weighted					−0.0333
377.4	0.944				−0.1091
178.6	0.742	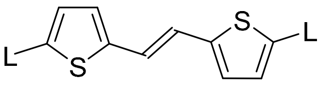	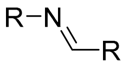	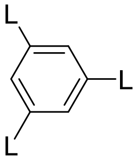	0.0506
254.2	0.320				0.0294
298.1	0.291	(Z)			−0.0901
173.7	0.275				0.3505
Weighted					−0.0433
363.5	0.931				0.0178
175.3	0.572	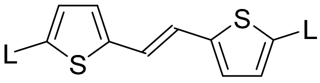	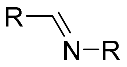	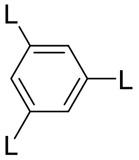	0.0574
182.0	0.327				−0.0161
162.9	0.319	(S)			0.0667
173.9	0.270				−0.0425
Weighted					0.0305
364.5	0.953				0.0184
175.2	0.538	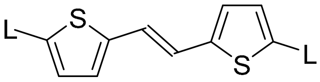	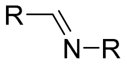	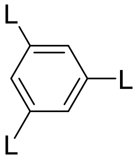	0.0299
179.0	0.325				0.2524
181.4	0.303	(Z)			0.0254
241.1	0.252				−0.0004
Weighted					0.0309
343.6	0.937				0.0003
275.7	0.546	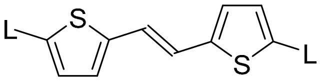	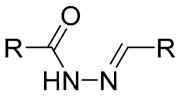	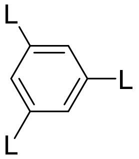	0.1243
182.1	0.364				0.1848
190.6	0.304	(S)			0.1003
170.4	0.252				−0.1065
Weighted					0.0422
344.1	0.939				0.0011
273.1	0.438	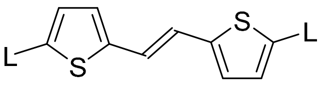	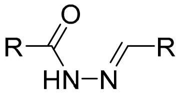	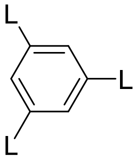	0.1126
181.3	0.412				0.1143
235.0	0.397	(Z)			0.0647
186.0	0.298				0.0058
Weighted					0.0341
361.1	0.949				−0.0172
177.5	0.417	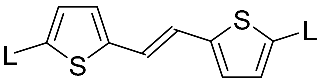	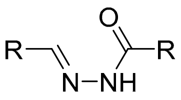	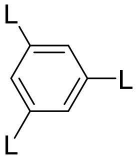	−0.0570
214.9	0.363				−0.2186
174.3	0.317	(S)			−0.0323
174.8	0.271				−0.0536
Weighted					−0.0618
362.0	0.957				−0.0162
177.4	0.529	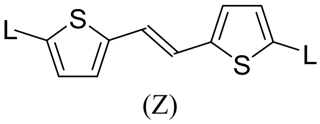	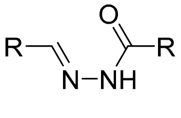	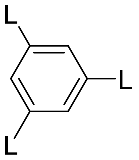	−0.0932
174.6	0.352				−0.1389
214.2	0.332	(Z)			−0.0354

172.7	0.320				−0.1428
Weighted					−0.0689
319.2	0.947	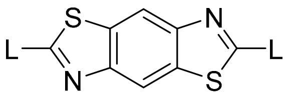 (S)	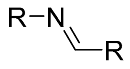	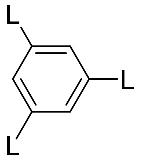	−0.0864
213.0	0.582				0.0026
289.4	0.472				−0.0458
171.0	0.427				−0.0500
217.3	0.410				−0.0252
Weighted					−0.0436
313.9	0.821	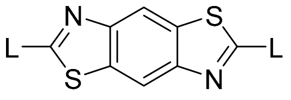 (Z)	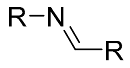	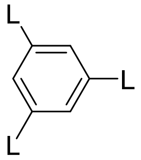	−0.0858
212.3	0.736				0.0090
164.5	0.635				0.2344
218.1	0.416				−0.0082
189.2	0.295				−0.2046
Weighted					−0.0433
287.2	0.910	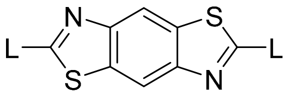 (S)	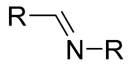	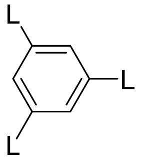	−0.0282
210.0	0.718				0.0399
334.4	0.303				0.1237
240.7	0.292				0.1311
184.4	0.252				−0.0548
Weighted					0.0223
285.8	0.808	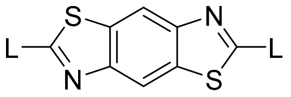 (Z)	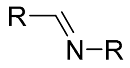	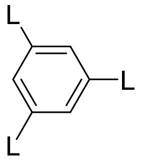	−0.0373
322.2	0.720				0.1207
211.8	0.528				0.0861
179.8	0.447				0.2136
210.7	0.435				0.1092
Weighted					0.0324
281.2	0.755	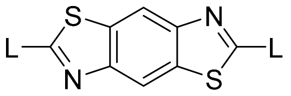 (S)	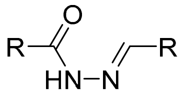	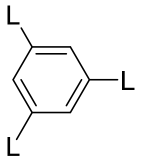	0.0911
208.7	0.664				0.0107
173.7	0.614				−0.0360
187.3	0.433				0.2922
182.1	0.391				0.1365
Weighted					0.0683
292.3	0.926	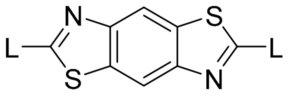 (Z)	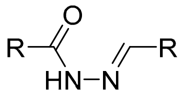	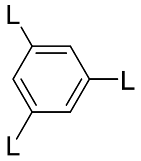	0.0568
297.7	0.618				0.0364
207.8	0.432				0.0039
232.2	0.346				0.1289
175.3	0.330				−0.0233
Weighted					0.0634
298.0	0.779	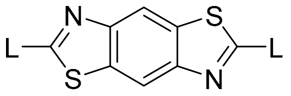 (S)	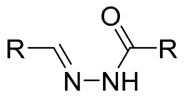	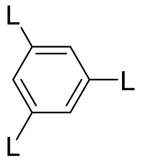	−0.0270
174.2	0.689				0.0866
295.5	0.609				−0.0203
172.3	0.534				0.0830

241.9	0.435				−0.0394
Weighted					−0.0162
294.1	0.884	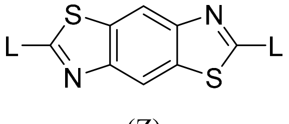 (Z)	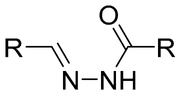	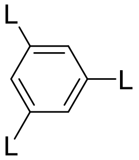	−0.0283
216.2	0.569				−0.0912
175.9	0.521				−0.0446
173.9	0.480				0.0723
172.1	0.463				0.0640
Weighted					−0.0348

[1] For certain systems (B02, B04, B05, B06, B07, B08), two near-coplanar conformers resulting from single-bond rotation between the ***UnitB*** and linker are identified by the symbols S and Z.

Table S2. Calculated net electron transfer ($\Delta q_{Ph\rightarrow UnitB}$) for the five excited states with the highest Light Harvesting Efficiency (LHE) among the first 50 states of model K02, along with the effective optical net electron transfer from ***Ph*** to ***UnitB*** ($Q_{Ph\rightarrow UnitB}$) for the first 50 excited states.

hv (nm)	LHE	***UnitB***[1]	Linker	***Ph***	$\Delta q_{Ph\rightarrow UnitB}$ / $Q_{Ph\rightarrow UnitB}$ (|*e*|)
313.7	0.675	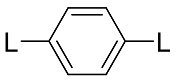	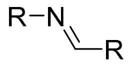	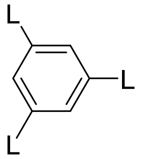	−0.1068
252.3	0.649				−0.0303
231.8	0.622				−0.0090
216.4	0.579				−0.1129
181.0	0.559				−0.0164
Weighted					−0.0503
268.7	0.790	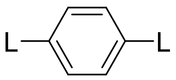	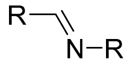	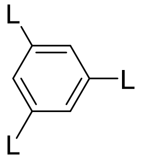	0.0196
311.2	0.615				0.1095
204.2	0.597				0.0366
179.5	0.582				0.1161
200.6	0.514				0.1230
Weighted					0.0566
258.1	0.919	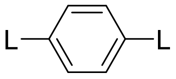	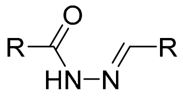	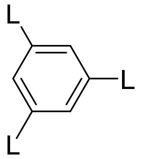	0.0031
274.9	0.721				0.0252
272.6	0.708				0.0428
282.1	0.490				0.0080
256.2	0.422				0.0020
Weighted					0.0189
303.4	0.974	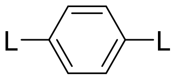	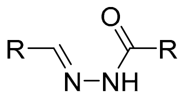	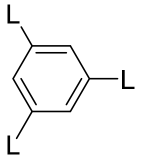	−0.0378
197.1	0.690				0.0002
195.3	0.606				−0.0425
236.4	0.556				−0.0060
198.3	0.496				−0.0341
Weighted					−0.0311
254.4	0.824	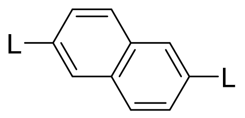 (S)	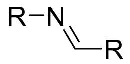	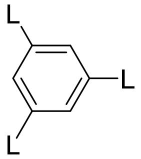	−0.0388
323.2	0.801				−0.1302
232.5	0.672				−0.0046
220.4	0.639				−0.1176
222.0	0.606				−0.0996
Weighted					−0.0627
249.3	0.857	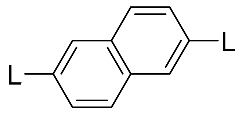 (Z)	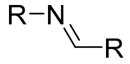	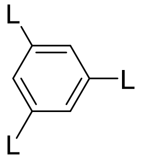	−0.0177
231.5	0.779				−0.0920
220.1	0.736				−0.1224
320.0	0.733				−0.1338
196.9	0.528				0.0760

Weighted					−0.0616
311.2	0.819	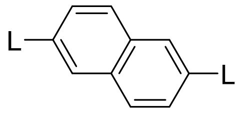 (S)	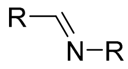	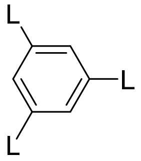	0.1076
245.6	0.817				0.0536
215.6	0.764				0.0932
278.9	0.676				0.0338
219.2	0.452				−0.0378
Weighted					0.0590
248.1	0.903	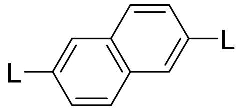 (Z)	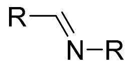	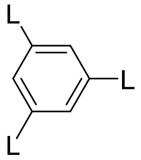	0.0616
323.2	0.795				0.0872
206.0	0.581				0.0701
230.6	0.529				0.1687
226.6	0.405				−0.0428
Weighted					0.0548
258.2	0.919	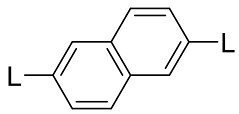 (S)	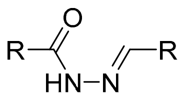	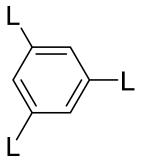	0.0046
287.5	0.731				0.0104
240.3	0.721				0.1067
221.6	0.710				0.0159
272.7	0.559				0.0366
Weighted					0.0243
258.2	0.918	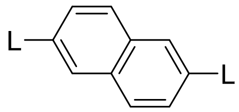 (Z)	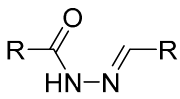	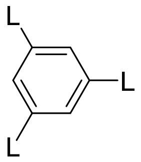	0.0045
289.6	0.747				−0.0053
238.6	0.688				0.0121
277.4	0.679				0.0223
216.9	0.563				0.1451
Weighted					0.0246
328.3	0.923	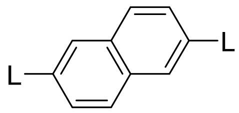 (S)	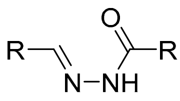	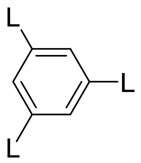	−0.0301
248.6	0.914				−0.0127
196.2	0.685				−0.0141
197.4	0.610				0.0444
236.5	0.601				−0.0075
Weighted					−0.0331
310.4	0.967	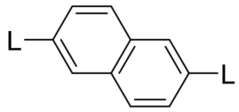 (Z)	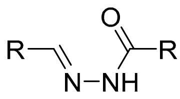	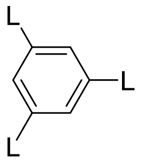	−0.0453
221.4	0.800				−0.1302
248.1	0.740				−0.0519
195.9	0.658				−0.0163
196.7	0.651				0.0212
Weighted					−0.0500
314.7	0.830	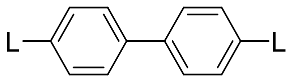	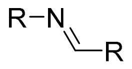	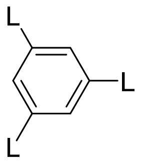	−0.1317
257.5	0.695				−0.0277
218.4	0.654				−0.1488
275.3	0.610				−0.0210
226.2	0.601				−0.0469

Weighted					−0.0814
280.0	0.905	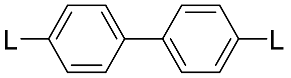	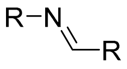	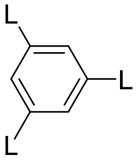	0.0024
310.1	0.844				0.1133
181.7	0.638				0.0178
203.9	0.621				0.0651
197.1	0.512				0.1470
Weighted					0.0447
258.1	0.919	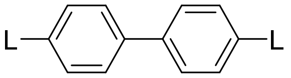	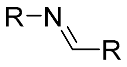	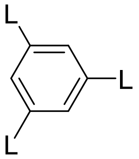	0.0032
275.1	0.900				0.0222
282.4	0.856				0.0181
181.9	0.660				0.0037
256.3	0.431				0.0041
Weighted					0.0168
305.1	0.993	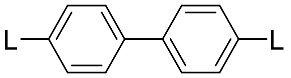	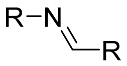	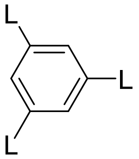	−0.0473
196.3	0.685				0.0117
197.2	0.651				0.0053
236.1	0.531				−0.0565
183.1	0.489				−0.0833
Weighted					−0.0457
311.6	0.852	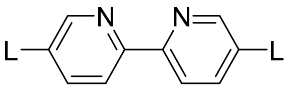 (S)	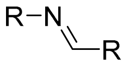	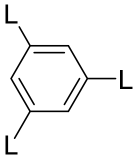	−0.1078
173.2	0.568				−0.0237
269.5	0.539				−0.0220
199.8	0.521				0.1043
214.1	0.499				−0.2365
Weighted					−0.0502
279.4	0.806	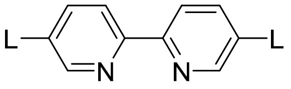 (Z)	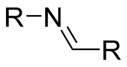	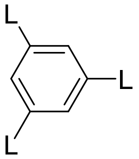	−0.0225
312.2	0.780				−0.1086
172.2	0.617				−0.0656
232.4	0.568				−0.1147
214.7	0.469				−0.2414
Weighted					−0.0560
281.0	0.812	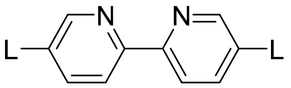 (S)	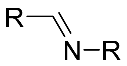	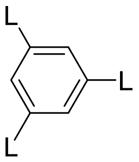	0.0373
313.5	0.788				0.1355
273.9	0.634				0.0699
173.7	0.479				−0.0053
175.2	0.465				−0.0216
Weighted					0.0651
286.7	0.827	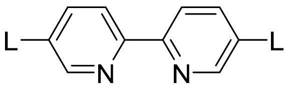 (Z)	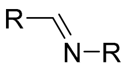	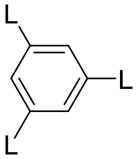	0.0150
316.4	0.706				0.1176
172.3	0.629				−0.0182
272.8	0.498				0.0140
212.3	0.424				0.0556

Weighted					0.0492
278.1	0.942	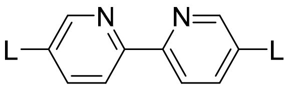	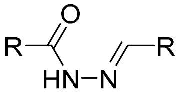	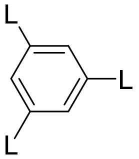	0.0647
258.2	0.917				0.0050
281.7	0.657				0.0152
256.4	0.488	(S)			0.0051
236.4	0.336				0.1978
Weighted					0.0384
279.4	0.936	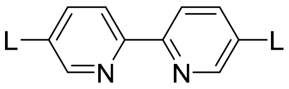	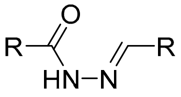	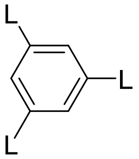	0.0658
258.3	0.914				0.0066
286.3	0.755				0.0132
256.4	0.495	(Z)			0.0051
251.0	0.326				0.0084
Weighted					0.0412
303.0	0.993	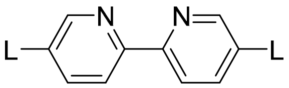	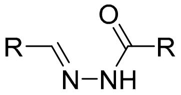	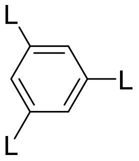	−0.0313
197.5	0.661				−0.0029
237.5	0.547				−0.0617
195.9	0.505	(S)			−0.0226
197.2	0.493				−0.0293
Weighted					−0.0342
291.7	0.966	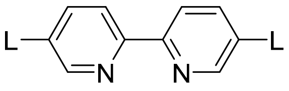	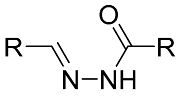	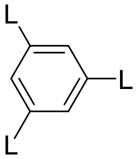	−0.0149
258.2	0.755				0.0001
255.9	0.740				−0.0016
288.6	0.612	(Z)			−0.0119
199.1	0.481				−0.0061
Weighted					0.0019
229.7	0.922	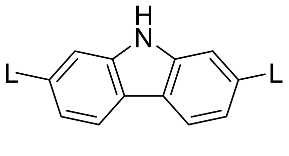	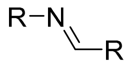	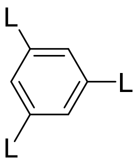	0.0011
326.8	0.879				−0.1441
221.0	0.757				−0.0712
214.1	0.629				−0.0275
281.6	0.459	(S)			−0.0393
Weighted					−0.0589
229.8	0.895	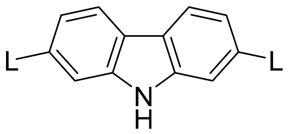	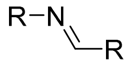	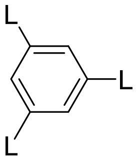	0.0152
328.8	0.871				−0.1437
221.2	0.747				−0.0792
215.2	0.673				−0.0098
279.8	0.522	(Z)			−0.0387
Weighted					−0.0535
221.8	0.850	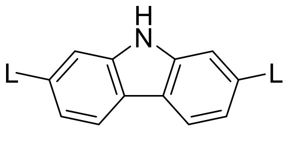	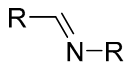	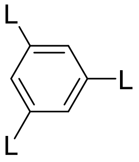	0.0498
317.5	0.842				0.0292
313.2	0.691				0.0209
289.7	0.534				0.0312
230.4	0.469	(S)			0.1234

Weighted					0.0203
219.5	0.935	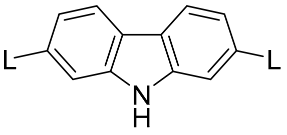	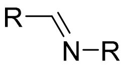	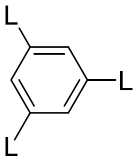	−0.0105
319.6	0.848				0.0357
313.7	0.651				0.0187
289.7	0.566				0.0322
175.4	0.500	(Z)			0.0045
Weighted					−0.0013
289.0	0.982	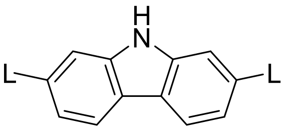	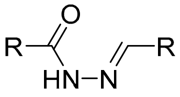	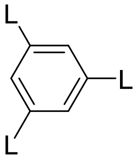	0.0132
258.1	0.919				0.0027
220.1	0.747				−0.0147
218.1	0.536				−0.0541
256.2	0.420	(S)			0.0015
Weighted					0.0021
288.5	0.974	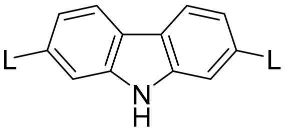	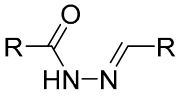	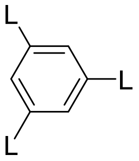	0.0117
258.1	0.920				0.0023
220.4	0.823				−0.0136
233.2	0.475				0.0421
277.3	0.465	(Z)			−0.0019
Weighted					0.0052
322.7	0.980	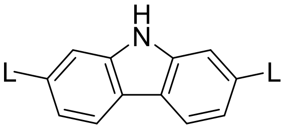	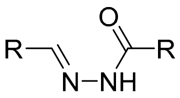	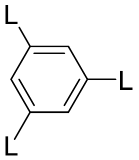	−0.0418
196.5	0.730				0.0113
223.4	0.699				−0.0579
196.0	0.610				0.0220
236.2	0.554	(S)			−0.1417
Weighted					−0.0547
323.7	0.974	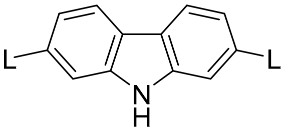	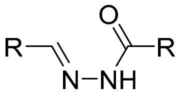	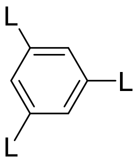	−0.0414
225.6	0.878				−0.0195
196.7	0.743				0.0092
196.1	0.658				0.0142
314.3	0.578	(Z)			−0.0228
Weighted					−0.0564
380.2	0.957	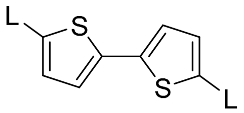	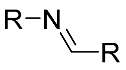	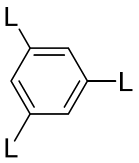	−0.0591
222.3	0.700				0.0463
220.3	0.500				−0.0928
207.8	0.439	(S)			0.1229
206.4	0.314				−0.0183
Weighted					−0.0328
368.4	0.939	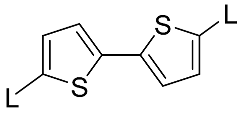	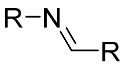	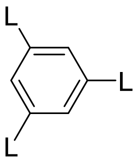	−0.1172
221.8	0.694				−0.0666
218.1	0.420				−0.0485
202.8	0.386				0.0796
203.3	0.348	(Z)			0.0894

Weighted					−0.0591
349.9	0.962	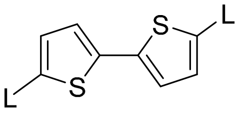 (S)	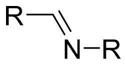	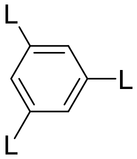	0.0352
198.4	0.406				0.1060
176.5	0.371				0.0406
213.3	0.361				0.0115
211.1	0.360				0.0274
Weighted					0.0318
354.4	0.951	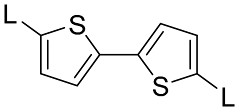 (Z)	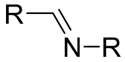	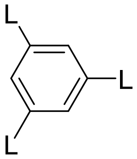	0.0347
218.4	0.502				0.0247
197.3	0.393				0.0929
201.3	0.307				−0.1443
213.5	0.304				0.0221
Weighted					0.0210
320.8	0.963	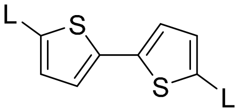 (S)	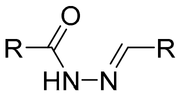	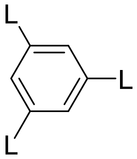	0.0140
258.5	0.907				0.0102
256.3	0.458				0.0033
243.6	0.364				0.1191
236.0	0.300				−0.0034
Weighted					0.0276
321.5	0.973	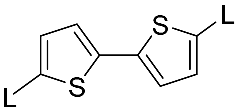 (Z)	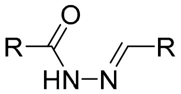	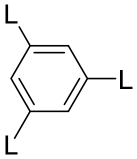	0.0101
258.6	0.909				0.0079
256.3	0.428				0.0019
241.2	0.364				0.0100
237.6	0.313				0.1900
Weighted					0.0241
360.0	0.979	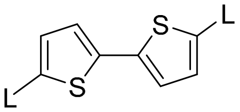 (S)	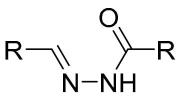	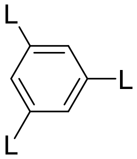	−0.0290
196.8	0.659				0.0003
197.1	0.642				0.0024
236.6	0.619				−0.0009
226.1	0.517				−0.0753
Weighted					−0.0297
365.5	0.971	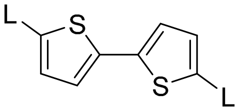 (Z)	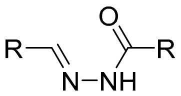	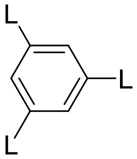	−0.0230
225.9	0.668				−0.1041
236.6	0.626				−0.0016
196.6	0.516				−0.0147
196.7	0.381				−0.0262
Weighted					−0.0365
407.3	0.976	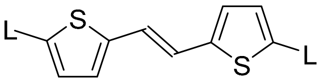 (S)	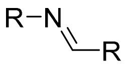	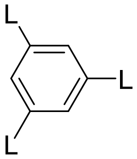	−0.0515
228.3	0.798				0.0041
210.2	0.658				0.1777
200.1	0.418				−0.0044
224.5	0.412				−0.0827

Weighted					−0.0301
399.3	0.973	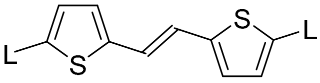	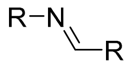	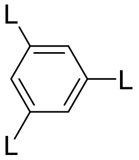	−0.0801
229.2	0.655				−0.0346
224.9	0.646				−0.0485
206.9	0.564	(Z)			0.2171
174.8	0.317				−0.1091
Weighted					−0.0491
382.9	0.982	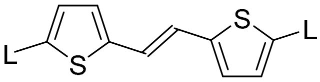	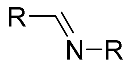	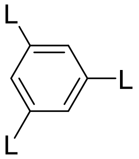	0.0184
199.3	0.441				0.0652
213.3	0.383				−0.0005
229.8	0.352	(S)			−0.0197
176.7	0.317				0.0105
Weighted					0.0143
384.0	0.973	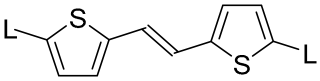	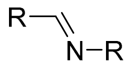	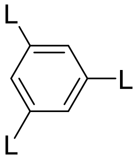	0.0186
226.2	0.540				−0.0095
199.2	0.481				0.0555
212.9	0.318	(Z)			0.0471
206.2	0.316				−0.0038
Weighted					0.0141
357.8	0.976	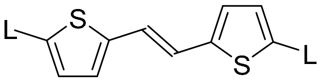	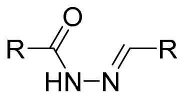	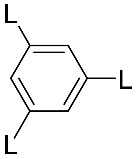	0.0059
259.3	0.860				0.0450
246.7	0.659				0.0618
256.5	0.551	(S)			0.0182
250.1	0.386				0.2810
Weighted					0.0338
357.3	0.982	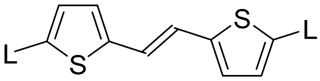	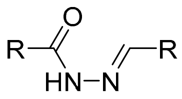	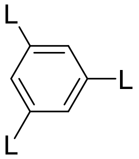	0.0048
259.0	0.891				0.0214
256.3	0.513				−0.0025
246.7	0.375	(Z)			0.0814
245.5	0.287				0.2671
Weighted					0.0169
392.6	0.988	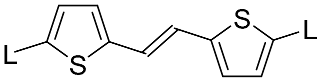	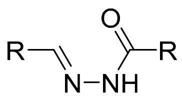	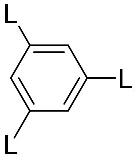	−0.0191
236.3	0.692				−0.0974
197.0	0.688				0.0401
236.7	0.590	(S)			−0.0027
196.5	0.454				0.1010
Weighted					−0.0250
394.0	0.984	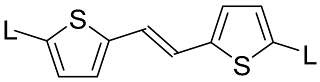	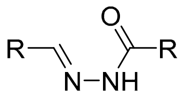	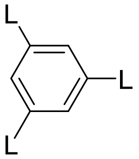	−0.0162
196.8	0.624				0.0263
236.7	0.611				−0.0017
235.8	0.590	(Z)			−0.0556
197.3	0.536				0.0950

Weighted					−0.0150
331.3	0.958	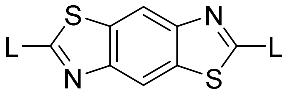 (S)	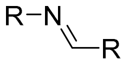	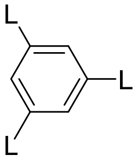	−0.0768
230.3	0.587				0.0075
206.1	0.582				0.0928
229.4	0.561				0.0140
216.5	0.471				−0.0618
Weighted					−0.0525
328.3	0.970	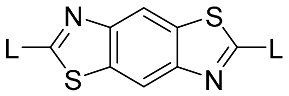 (Z)	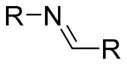	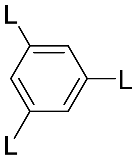	−0.0823
232.5	0.650				0.0183
200.9	0.540				−0.1915
205.8	0.515				0.0870
211.5	0.467				−0.2105
Weighted					−0.0729
301.1	0.854	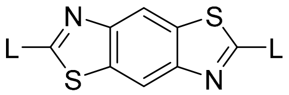 (S)	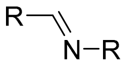	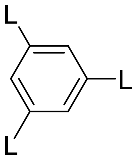	0.0034
338.5	0.777				0.1286
215.7	0.731				0.0279
230.0	0.622				0.0239
199.4	0.434				0.0886
Weighted					0.0449
329.6	0.848	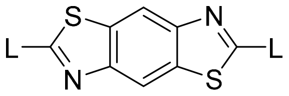 (Z)	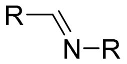	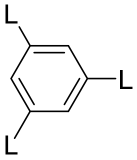	0.0980
218.8	0.812				0.0132
297.4	0.735				0.0156
207.3	0.400				0.2065
227.7	0.399				0.0175
Weighted					0.0395
258.5	0.902	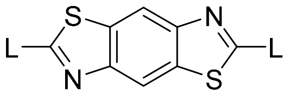 (S)	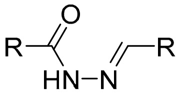	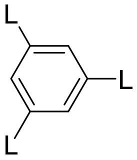	0.0125
293.3	0.840				0.0191
284.9	0.705				0.0461
213.1	0.674				0.0308
238.7	0.638				0.1227
Weighted					0.0348
302.5	0.945	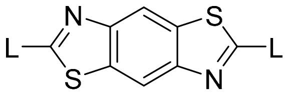 (Z)	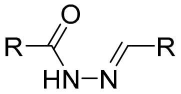	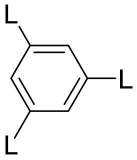	0.0668
259.8	0.850				0.0416
309.6	0.668				0.0163
213.0	0.506				0.0726
241.3	0.501				0.1337
Weighted					0.0486
324.5	0.986	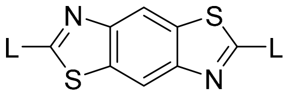 (S)	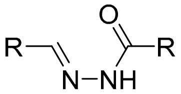	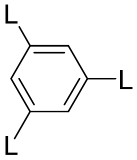	−0.0275
238.0	0.546				−0.1229
198.7	0.536				−0.0360
237.3	0.481				−0.0182
240.3	0.474				−0.1627

Weighted					−0.0531
325.5	0.920	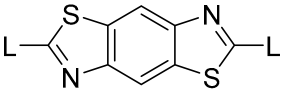 (Z)	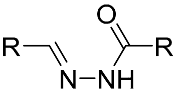	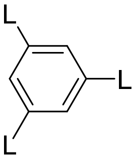	−0.0219
316.7	0.873				−0.0271
236.7	0.626				−0.0838
220.3	0.595				−0.1032
221.2	0.482				−0.0489
Weighted					−0.0476

[1] For certain systems (B02, B04, B05, B06, B07, B08), two near-coplanar conformers resulting from single-bond rotation between the ***UnitB*** and linker are identified by the symbols S and Z.

Table S3. Calculated net electron transfer ($\Delta q_{Ph \to UnitB}$) for the five excited states with the highest Light Harvesting Efficiency (LHE) among the first 50 states of model K03, along with the effective optical net electron transfer from ***Ph*** to ***UnitB*** **(**$Q_{Ph \to UnitB}$) for the first 50 excited states.

hv (nm)	LHE	***UnitB***[1]	Linker	***Ph***	$\Delta q_{Ph \to UnitB}$ / $Q_{Ph \to UnitB}$ (|*e*|)
252.7	0.882	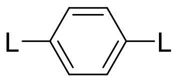	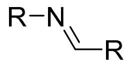	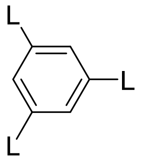	−0.0471
252.6	0.881				−0.0474
175.8	0.662				0.0166
175.8	0.661				0.0167
304.9	0.570				−0.1166
Weighted					−0.0707
256.5	0.861	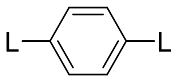	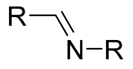	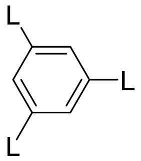	0.0388
256.5	0.861				0.0393
186.8	0.627				0.0185
186.8	0.627				0.0199
299.8	0.599				0.1072
Weighted					0.0647
269.6	0.949	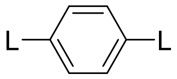	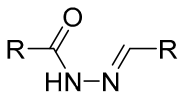	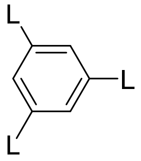	0.0417
269.6	0.949				0.0418
177.5	0.471				−0.0012
177.6	0.440				−0.0075
278.3	0.404				−0.0015
Weighted					0.0303
274.0	0.834	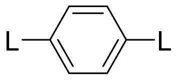	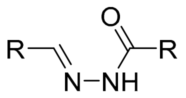	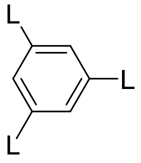	−0.0585
273.9	0.834				−0.0584
280.4	0.723				−0.0287
280.4	0.722				−0.0288
201.5	0.512				−0.1097
Weighted					−0.0859
249.6	0.848	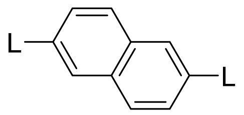 (S)	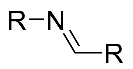	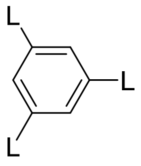	−0.2121
250.0	0.847				−0.2095
319.1	0.813				−0.1539
319.6	0.808				−0.1534
200.3	0.694				0.0038
Weighted					−0.1162
246.0	0.883	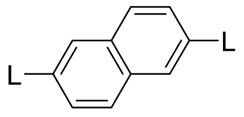 (Z)	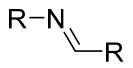	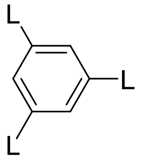	−0.1802
246.2	0.880				−0.1865
317.2	0.758				−0.1651
200.6	0.753				−0.0011
317.0	0.741				−0.1629

Weighted					−0.1245
243.6	0.900	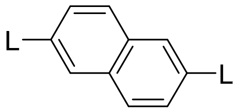	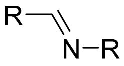	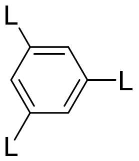	0.1274
244.3	0.876				0.1253
305.2	0.843				0.1182
312.0	0.757	(S)			0.1188
269.4	0.693				0.0050
Weighted					0.0739
245.2	0.923	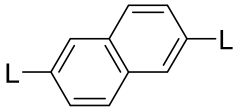	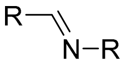	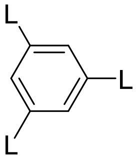	0.1154
245.7	0.911				0.1240
312.6	0.816				0.1145
314.0	0.807				0.1171
192.8	0.694	(Z)			0.0169
Weighted					0.0700
282.2	0.821	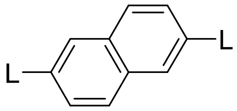	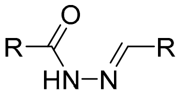	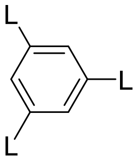	0.0112
281.9	0.817				0.0091
271.7	0.774				0.0146
272.0	0.755				0.0128
224.4	0.673	(S)			0.1736
Weighted					0.0120
273.7	0.855	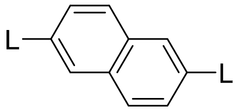	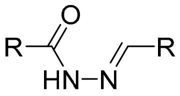	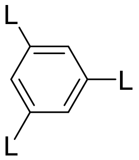	0.0217
274.7	0.851				0.0180
282.9	0.829				−0.0003
282.6	0.820				−0.0018
222.1	0.648	(Z)			0.1496
Weighted					0.0155
303.5	0.913	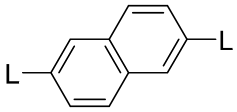	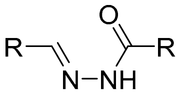	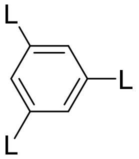	−0.0782
303.7	0.901				−0.0773
236.8	0.753				−0.2189
237.0	0.734				−0.2073
259.8	0.703	(S)			−0.1825
Weighted					−0.1202
293.7	0.944	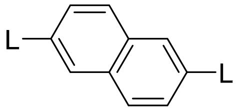	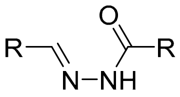	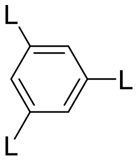	−0.0988
293.8	0.941				−0.1003
219.9	0.623				−0.2877
219.7	0.610				−0.2944
235.7	0.581	(Z)			−0.2622
Weighted					−0.1442
263.4	0.923	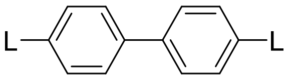	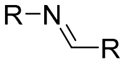	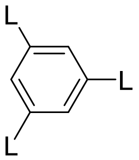	−0.0604
263.0	0.919				−0.0597
316.6	0.883				−0.1471
316.9	0.879				−0.1469
201.0	0.349				−0.1866

Weighted					−0.1167
273.0	0.935	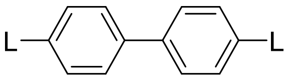	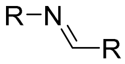	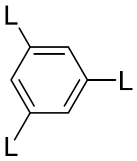	−0.0146
273.5	0.930				−0.0121
307.5	0.909				0.1178
308.9	0.905				0.1175
199.0	0.617				−0.0020
Weighted					0.0441
281.4	0.931	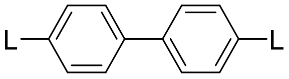	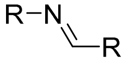	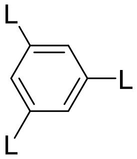	0.0125
281.3	0.931				0.0114
274.9	0.926				0.0219
274.7	0.924				0.0224
192.6	0.481				0.0157
Weighted					0.0224
295.2	0.992	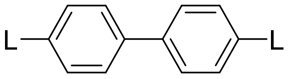	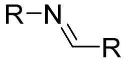	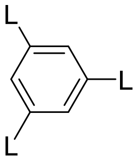	−0.0882
295.3	0.992				−0.0877
198.3	0.551				−0.0570
198.3	0.548				−0.0577
209.3	0.415				−0.0832
Weighted					−0.0997
314.4	0.894	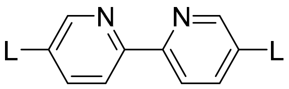 (S)	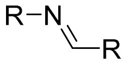	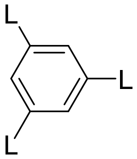	−0.1271
314.4	0.893				−0.1271
260.8	0.719				−0.0394
260.8	0.717				−0.0388
270.5	0.511				−0.0310
Weighted					−0.0842
274.6	0.865	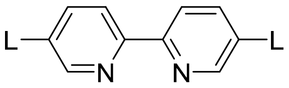 (Z)	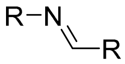	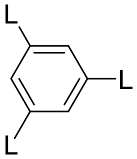	−0.0472
274.7	0.865				−0.0474
314.7	0.805				−0.1249
314.7	0.804				−0.1245
259.4	0.484				−0.0174
Weighted					−0.0754
309.7	0.884	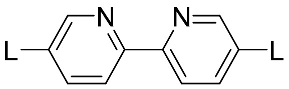 (S)	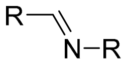	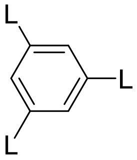	0.1292
309.7	0.884				0.1292
271.6	0.852				−0.0038
271.6	0.849				−0.0039
277.9	0.526				−0.0163
Weighted					0.0564
282.4	0.876	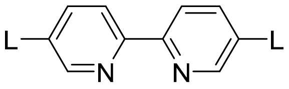 (Z)	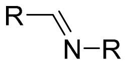	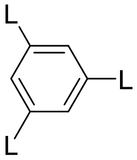	0.0018
282.3	0.875				0.0019
312.7	0.822				0.1076
312.7	0.822				0.1082
268.4	0.479				−0.0076

Weighted					0.0472
277.7	0.989	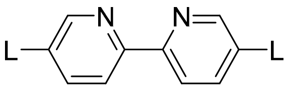 (S)	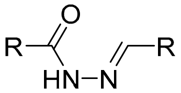	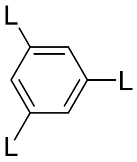	0.0544
277.7	0.989				0.0544
221.6	0.383				0.1026
221.6	0.382				0.1020
243.5	0.336				−0.0179
Weighted					0.0492
278.6	0.977	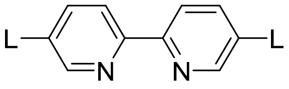 (Z)	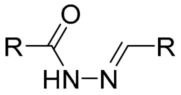	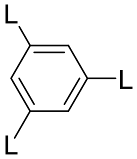	0.0487
278.6	0.976				0.0483
284.7	0.665				0.0015
284.7	0.653				0.0015
242.7	0.406				0.0020
Weighted					0.0439
292.4	0.992	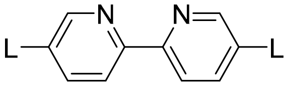 (S)	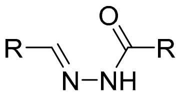	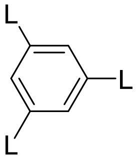	−0.0669
292.4	0.992				−0.0670
199.5	0.455				−0.0393
199.5	0.455				−0.0396
231.9	0.199				−0.0789
Weighted					−0.0635
291.8	0.985	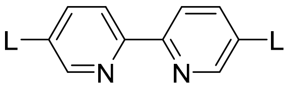 (Z)	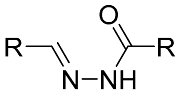	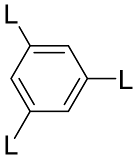	−0.0657
292.0	0.982				−0.0634
285.5	0.591				−0.0388
285.9	0.525				−0.0374
201.2	0.376				−0.0756
Weighted					−0.0678
325.9	0.922	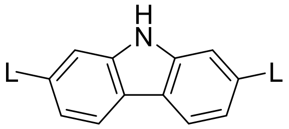 (S)	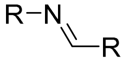	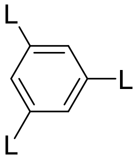	−0.1676
325.9	0.921				−0.1675
269.1	0.811				−0.0687
269.1	0.810				−0.0696
226.4	0.739				−0.0157
Weighted					−0.1011
328.6	0.911	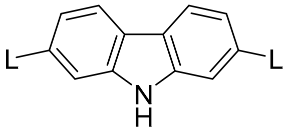 (Z)	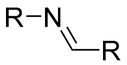	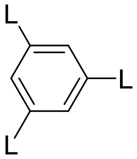	−0.1678
328.7	0.911				−0.1677
267.8	0.776				−0.1215
267.8	0.776				−0.1217
232.5	0.682				−0.0086
Weighted					−0.1123
311.6	0.968	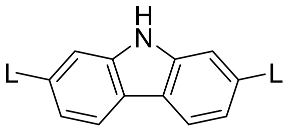 (S)	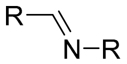	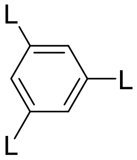	0.0908
311.7	0.968				0.0911
281.2	0.710				0.0006
281.2	0.709				0.0005
225.1	0.694				−0.0874

Weighted					0.0249
315.1	0.961	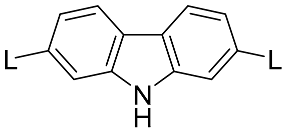	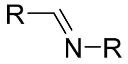	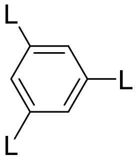	0.0837
315.0	0.960				0.0835
228.2	0.689				−0.0610
228.3	0.685				−0.0614
211.3	0.679	(Z)			−0.0059
Weighted					0.0252
285.7	0.991	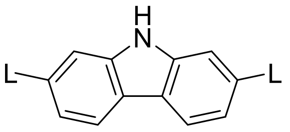	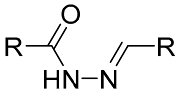	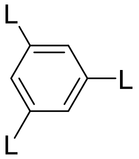	0.0085
285.9	0.990				0.0073
221.6	0.830				−0.0142
221.6	0.814				−0.0125
274.9	0.591	(S)			−0.0016
Weighted					−0.0260
285.5	0.984	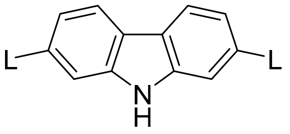	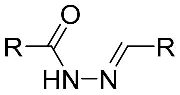	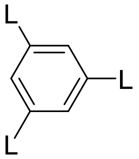	0.0069
285.5	0.984				0.0066
221.9	0.769				0.0251
221.9	0.768				0.0253
275.3	0.715	(Z)			0.0028
Weighted					−0.0096
305.7	0.989	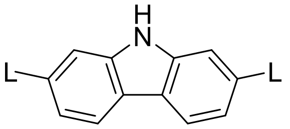	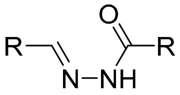	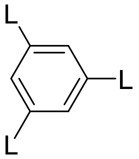	−0.1025
305.6	0.989				−0.1023
226.8	0.808				−0.0415
226.8	0.808				−0.0417
220.3	0.557	(S)			−0.0534
Weighted					−0.0951
310.7	0.963	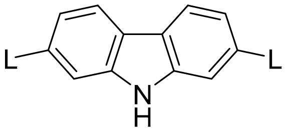	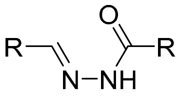	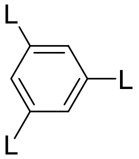	−0.0774
310.7	0.962				−0.0771
305.1	0.745				−0.0506
305.1	0.744				−0.0508
228.3	0.699	(Z)			−0.0502
Weighted					−0.0727
368.4	0.979	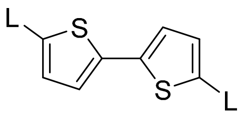	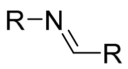	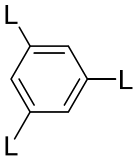	−0.1046
368.7	0.979				−0.1036
234.9	0.334				−0.0693
234.9	0.326	(S)			−0.0691
204.7	0.217				−0.0101
Weighted					−0.0892
365.6	0.962	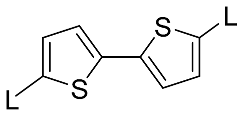	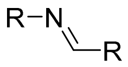	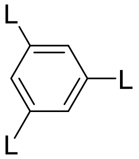	−0.1609
365.4	0.961				−0.1619
279.7	0.445				−0.0697
279.8	0.434	(Z)			−0.0700
234.3	0.341				−0.0329

Weighted					−0.1228
340.8	0.976	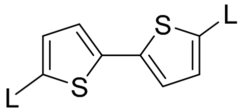 (S)	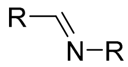	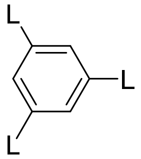	0.0426
340.6	0.976				0.0427
204.9	0.352				0.0950
204.9	0.351				0.0940
230.6	0.213				−0.0274
Weighted					0.0461
343.8	0.974	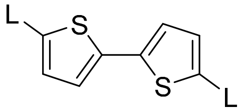 (Z)	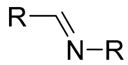	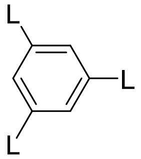	0.0390
343.9	0.974				0.0389
203.5	0.398				0.0662
203.5	0.397				0.0654
231.6	0.381				−0.0007
Weighted					0.0341
310.3	0.983	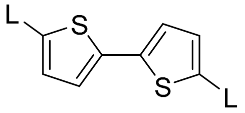 (S)	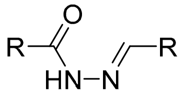	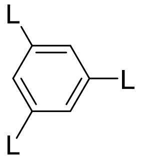	0.0105
310.1	0.983				0.0108
267.7	0.455				0.0685
267.7	0.452				0.0687
229.0	0.325				0.0023
Weighted					0.0150
312.2	0.987	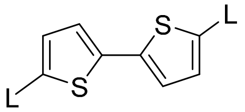 (Z)	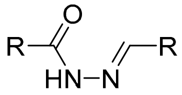	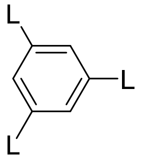	0.0038
312.2	0.986				0.0036
266.7	0.389				0.0621
266.7	0.388				0.0616
222.7	0.349				0.0989
Weighted					0.0109
337.4	0.983	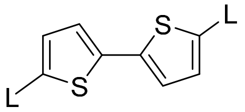 (S)	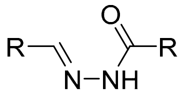	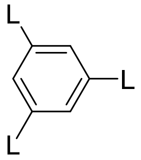	−0.0702
337.2	0.982				−0.0701
228.6	0.195				−0.0529
228.6	0.182				−0.0517
233.3	0.170				−0.0521
Weighted					−0.0776
339.2	0.977	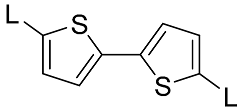 (Z)	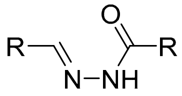	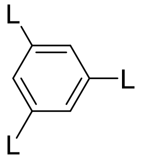	−0.0620
339.5	0.977				−0.0618
232.0	0.204				−0.0462
232.0	0.189				−0.0461
226.5	0.183				−0.0881
Weighted					−0.0736
402.3	0.990	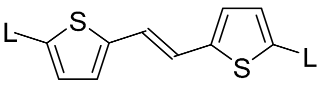 (S)	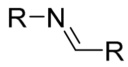	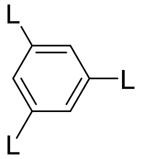	−0.0942
402.3	0.990				−0.0942
214.7	0.388				−0.0928
214.7	0.387				−0.0946
257.9	0.314				−0.0778

Weighted	-0.084				−0.1271
396.4	0.986	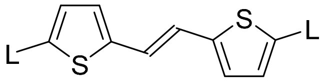	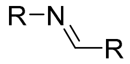	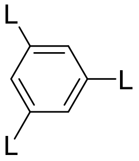	−0.1387
396.3	0.985				−0.1397
301.2	0.399				−0.1326
301.5	0.397	(Z)			−0.1300
254.8	0.382				−0.1203
Weighted					−0.1271
376.2	0.991	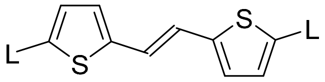	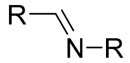	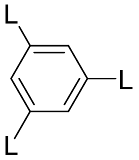	0.0178
375.8	0.991				0.0179
348.3	0.469				0.0050
248.5	0.207	(S)			0.0119
248.3	0.204				0.0137
Weighted					0.0197
376.9	0.988	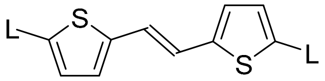	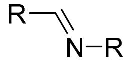	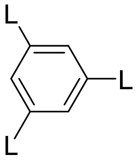	0.0201
376.8	0.988				0.0204
349.8	0.344				0.0126
208.2	0.308	(Z)			0.1489
208.2	0.308				0.1488
Weighted					0.0275
347.6	0.989	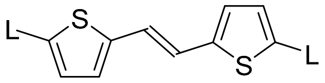	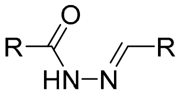	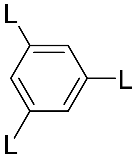	0.0004
347.6	0.989				0.0001
278.7	0.584				0.0989
278.5	0.577	(S)			0.0970
242.4	0.355				0.0243
Weighted					0.0187
348.7	0.992	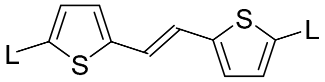	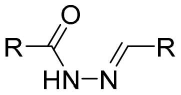	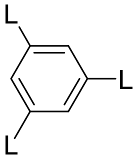	−0.0015
348.6	0.992				−0.0024
276.6	0.543				0.0930
276.5	0.537	(Z)			0.0907
238.3	0.293				0.0796
Weighted					0.0101
371.6	0.993	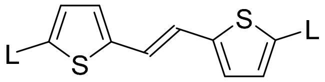	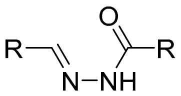	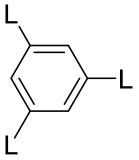	−0.0450
371.6	0.993				−0.0451
221.6	0.287				−0.1937
221.6	0.286	(S)			−0.1926
240.0	0.224				−0.0182
Weighted					−0.0660
371.8	0.989	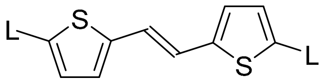	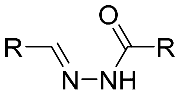	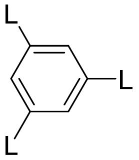	−0.0392
371.8	0.989				−0.0390
356.7	0.416				−0.0326
222.0	0.320	(Z)			−0.2315
222.0	0.319				−0.2308

Weighted					−0.0625
329.0	0.979	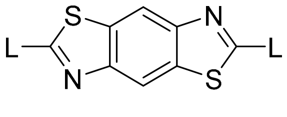 (S)	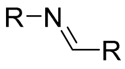	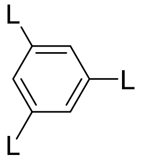	−0.1250
329.2	0.979				−0.1267
211.9	0.814				0.0008
212.0	0.804				0.0032
226.3	0.540				−0.0141
Weighted					−0.0862
327.7	0.983	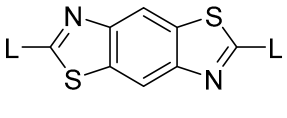 (Z)	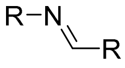	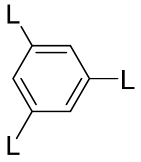	−0.1314
327.7	0.982				−0.1280
211.5	0.733				−0.0240
211.5	0.727				−0.0238
225.7	0.493				−0.0305
Weighted					−0.0948
293.0	0.888	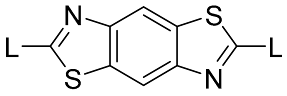 (S)	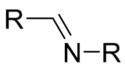	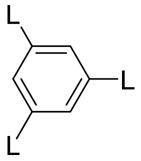	−0.0245
293.3	0.888				−0.0246
331.9	0.869				0.1216
332.0	0.866				0.1210
209.8	0.830				0.0265
Weighted					0.0199
322.4	0.937	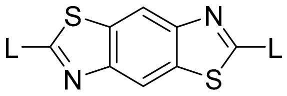 (Z)	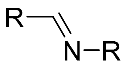	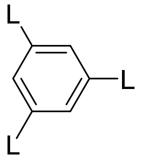	0.0973
322.3	0.937				0.0987
210.8	0.849				0.0191
210.8	0.849				0.0183
289.5	0.747				−0.0223
Weighted					0.0271
285.2	0.985	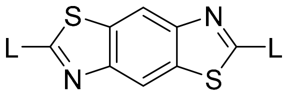 (S)	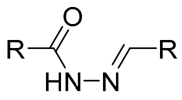	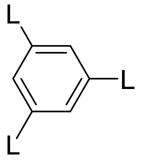	0.0592
285.0	0.984				0.0585
225.7	0.785				0.1014
225.7	0.784				0.1004
208.6	0.707				0.0086
Weighted					0.0495
301.0	0.968	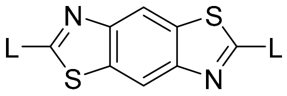 (Z)	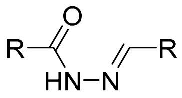	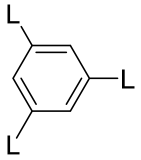	0.0551
300.9	0.968				0.0549
230.5	0.730				0.0753
230.5	0.729				0.0749
294.6	0.609				0.0015
Weighted					0.0452
307.7	0.982	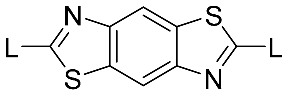 (S)	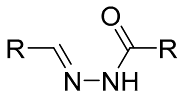	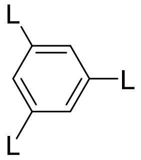	−0.0637
307.6	0.982				−0.0639
230.4	0.581				−0.1265
230.4	0.581				−0.1263
301.6	0.450				−0.0411

Weighted					−0.0872
303.8	0.963	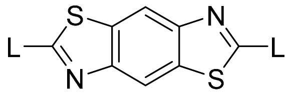 (Z)	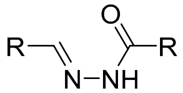	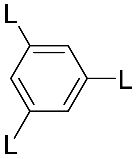	−0.0671
303.8	0.963				−0.0670
309.2	0.737				−0.0458
309.2	0.736				−0.0457
218.7	0.511				−0.1100
Weighted					−0.0720

[1] For certain systems (B02, B04, B05, B06, B07, B08), two near-coplanar conformers resulting from single-bond rotation between the ***UnitB*** and linker are identified by the symbols S and Z.

Table S4. Calculated net electron transfer ($\Delta q_{Ph \to UnitB}$) for the five excited states with the highest Light Harvesting Efficiency (LHE) among the first 50 states of model K04, along with the effective optical net electron transfer from ***Ph*** to ***UnitB*** **(**$Q_{Ph \to UnitB}$) for the first 50 excited states.

hv (nm)	LHE	***UnitB***[1]	Linker	***Ph***	$\Delta q_{Ph \to UnitB}$ / $Q_{Ph \to UnitB}$ (|*e*|)
260.4	0.880	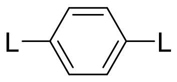	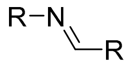	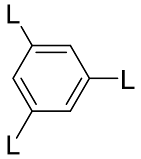	−0.0107
327.1	0.839				−0.1092
182.3	0.758				0.0130
183.7	0.744				−0.0881
201.5	0.489				−0.2053
Weighted					−0.0620
322.4	0.858	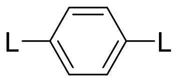	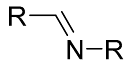	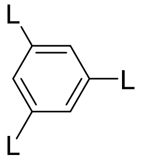	0.1151
182.4	0.854				−0.0064
275.4	0.808				−0.0067
205.3	0.586				0.1417
178.2	0.479				−0.0658
Weighted					0.0504
276.1	0.909	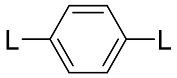	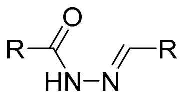	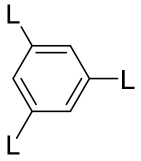	0.0536
181.4	0.828				0.0927
284.6	0.817				0.0205
186.3	0.577				0.2400
200.0	0.445				0.1811
Weighted					0.0711
305.1	0.980	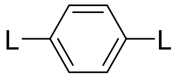	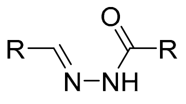	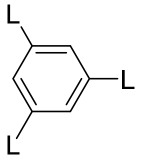	−0.0261
177.4	0.852				0.0043
207.7	0.535				0.0298
172.3	0.403				−0.0631
205.7	0.387				−0.0293
Weighted					−0.0180
257.5	0.928	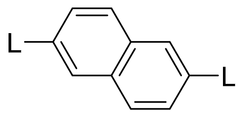 (S)	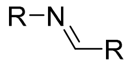	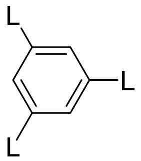	−0.0329
335.2	0.907				−0.1282
206.8	0.822				−0.1834
177.5	0.530				0.0544
268.8	0.513				−0.0498
Weighted					−0.0722
251.0	0.967	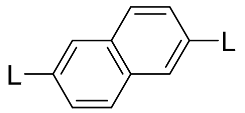 (Z)	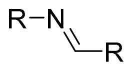	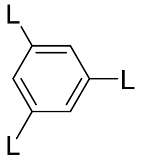	0.0037
330.2	0.865				−0.1330
206.2	0.780				−0.1319
179.6	0.468				0.1482
199.8	0.466				−0.2900

Weighted					−0.0631
321.4	0.936	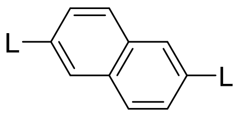	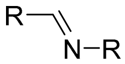	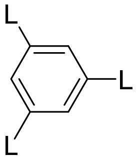	0.1008
246.7	0.833				0.0626
213.6	0.755				0.1351
171.3	0.682	(S)			−0.0953
280.2	0.650				0.0049
Weighted					0.0795
248.3	0.964	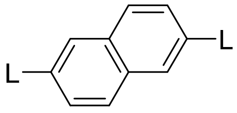	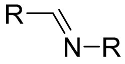	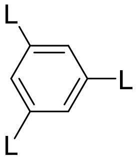	0.0577
335.2	0.898				0.0758
209.9	0.568				0.0819
176.4	0.515	(Z)			0.0371
173.5	0.496				0.0196
Weighted					0.0628
290.7	0.901	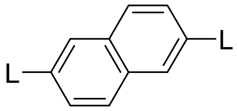	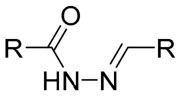	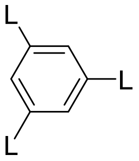	0.0332
275.4	0.732				0.0302
217.6	0.685				0.0184
258.5	0.668	(S)			0.0113
235.5	0.648				0.2130
Weighted					0.0764
292.3	0.890	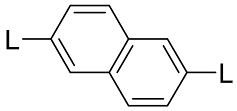	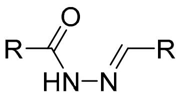	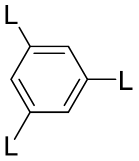	0.0056
216.4	0.852				0.0602
277.7	0.677				0.0191
282.5	0.618	(Z)			0.0231
263.9	0.617				0.0181
Weighted					0.0509
256.6	0.954	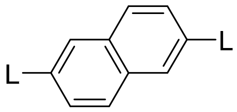	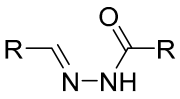	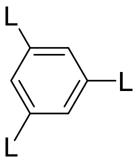	−0.0699
329.0	0.940				−0.0235
174.6	0.628				−0.0831
211.8	0.501	(S)			−0.0320
174.9	0.496				−0.1856
Weighted					−0.0610
310.9	0.978	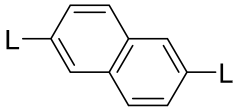	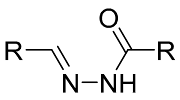	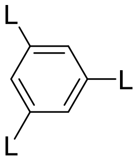	−0.0346
216.4	0.864				−0.1433
252.1	0.775				−0.0307
172.7	0.574	(Z)			0.0784
175.2	0.475				−0.0393
Weighted					−0.0508
322.4	0.933	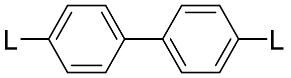	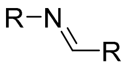	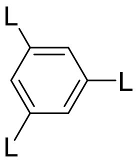	−0.1231
184.2	0.921				−0.0338
264.8	0.906				−0.0238
199.9	0.477				−0.2905
182.8	0.475				−0.1169

Weighted					−0.0728
316.8	0.953	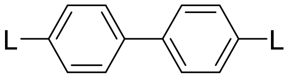	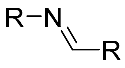	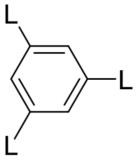	0.1117
281.7	0.845				−0.0063
183.0	0.789				0.0274
188.6	0.580				−0.0784
204.0	0.463				0.0770
Weighted					0.0563
285.2	0.969	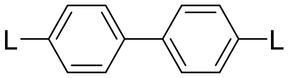	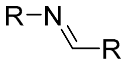	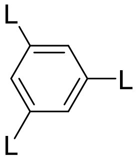	0.0200
275.1	0.901				0.0248
182.6	0.811				−0.0018
184.1	0.568				0.0384
227.0	0.507				0.2109
Weighted					0.0706
305.5	0.995	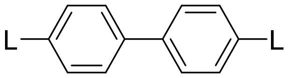	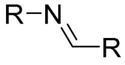	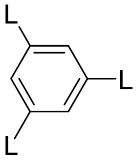	−0.0302
184.3	0.838				0.0085
180.4	0.554				−0.0759
174.0	0.538				0.0379
171.8	0.463				−0.1603
Weighted					−0.0296
320.2	0.931	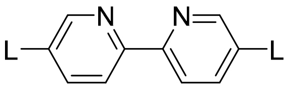	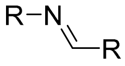	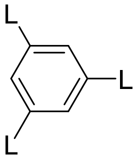	−0.1084
276.2	0.706				−0.0339
262.8	0.546				−0.0268
181.2	0.520				0.0280
197.6	0.430				−0.1616
Weighted					−0.0486
321.1	0.909	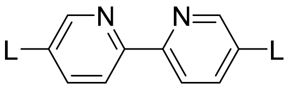	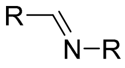	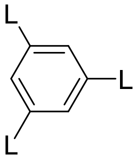	0.1314
285.6	0.809				−0.0003
180.5	0.622				0.1598
173.5	0.588				−0.1332
173.9	0.350				−0.1579
Weighted					0.0730
280.9	0.958	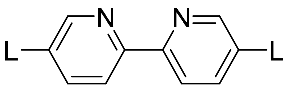	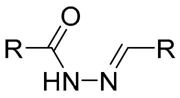	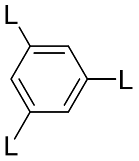	0.0567
286.5	0.743				0.0103
232.3	0.475				0.1881
181.5	0.475				0.0931
289.9	0.467				−0.0029
Weighted					0.0675
304.3	0.995	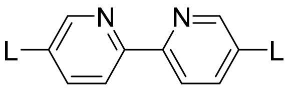	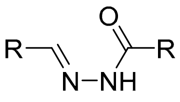	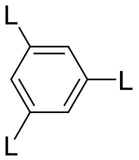	−0.0207
176.8	0.677				0.0088
175.5	0.573				0.0871
182.6	0.384				−0.1381
185.4	0.334				0.1841

Weighted					−0.0068
337.1	0.944	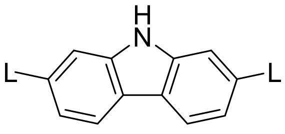 (S)	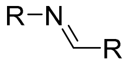	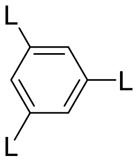	−0.1361
229.8	0.723				−0.0216
271.4	0.604				−0.0658
257.8	0.578				−0.2582
212.0	0.544				−0.1310
Weighted					−0.0993
328.1	0.962	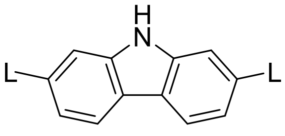 (S)	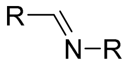	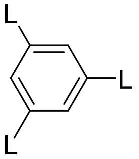	0.0558
228.6	0.847				0.1046
175.0	0.695				−0.0987
215.7	0.563				0.1859
319.4	0.436				−0.0411
Weighted					0.0319
292.6	0.988	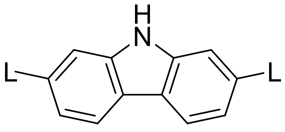 (S)	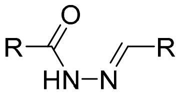	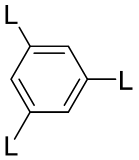	0.0188
219.1	0.844				−0.0328
180.0	0.501				0.1566
257.9	0.485				0.0256
276.1	0.458				0.0010
Weighted					0.0363
323.3	0.985	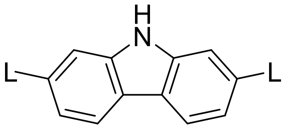 (S)	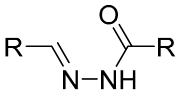	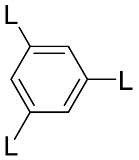	−0.0296
220.6	0.674				−0.1504
174.1	0.620				0.1085
227.8	0.487				−0.1290
313.4	0.446				−0.0220
Weighted					−0.0536
401.9	0.978	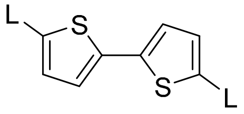 (S)	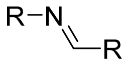	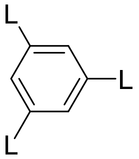	−0.0610
177.7	0.743				−0.0730
242.3	0.543				−0.1125
237.1	0.419				0.0436
215.4	0.366				−0.2373
Weighted					−0.0736
385.2	0.968	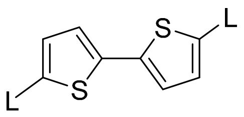 (Z)	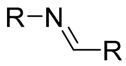	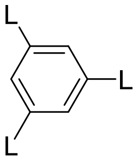	−0.1182
236.6	0.552				−0.0205
181.1	0.455				−0.0219
180.8	0.377				0.0153
243.0	0.376				−0.1653
Weighted					−0.0725
363.0	0.979	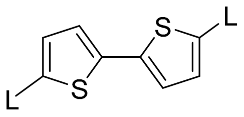 (S)	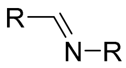	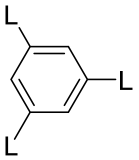	0.0414
176.9	0.716				0.0492
180.3	0.372				−0.0834
179.1	0.346				−0.0071
231.7	0.301				0.0206

Weighted					0.0377
367.1	0.969	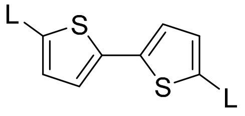 (Z)	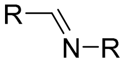	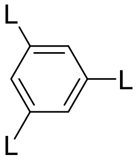	0.0443
177.9	0.794				0.0117
224.1	0.518				0.0982
229.7	0.438				0.2058
173.1	0.376				0.0982
Weighted					0.0428
324.1	0.977	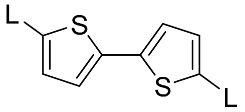 (S)	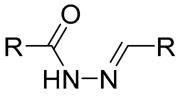	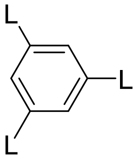	0.0261
182.9	0.620				0.1453
268.2	0.522				0.1168
239.3	0.460				0.0199
237.6	0.446				0.1297
Weighted					0.0765
326.5	0.983	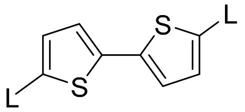 (Z)	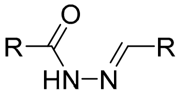	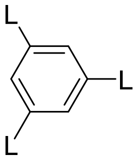	0.0192
181.9	0.663				0.1610
265.5	0.581				0.1072
190.5	0.524				0.1702
233.7	0.485				0.1579
Weighted					0.0719
360.5	0.983	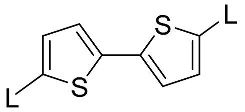 (S)	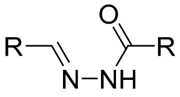	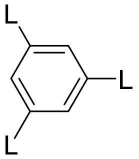	−0.0230
223.2	0.658				−0.0715
177.2	0.640				−0.1752
256.1	0.352				−0.1582
175.1	0.324				0.0290
Weighted					−0.0541
366.4	0.976	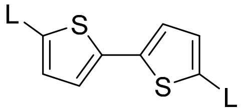 (Z)	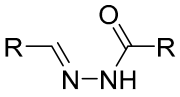	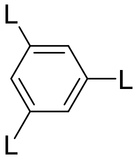	−0.0186
175.2	0.729				−0.0382
177.5	0.691				−0.1704
222.6	0.645				−0.0616
198.2	0.323				−0.1558
Weighted					−0.0620
361.0	0.982	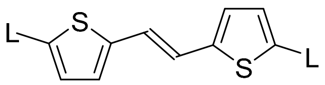 (S)	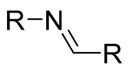	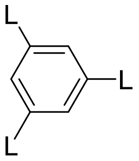	0.0072
245.5	0.769				0.1397
193.4	0.630				0.0513
278.9	0.568				0.1751
184.9	0.444				0.1529
Weighted					−0.0461
361.1	0.987	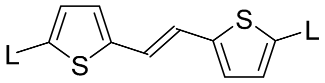 (Z)	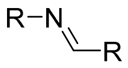	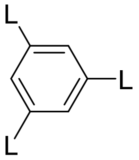	0.0082
273.0	0.642				0.1581
192.6	0.596				0.1063
183.6	0.501				0.1388
239.2	0.417				0.1434

Weighted					−0.0657
394.9	0.989	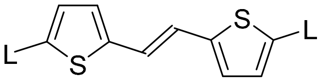	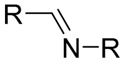	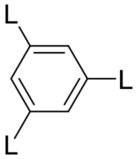	0.0217
178.4	0.721				0.1041
236.5	0.527				0.0165
171.6	0.299	(S)			−0.0800
174.1	0.269				0.0556
Weighted					0.0340
396.0	0.982	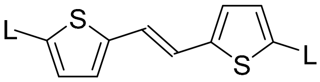	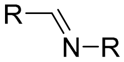	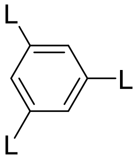	0.0242
177.7	0.868				0.0436
231.7	0.709				0.0053
171.9	0.445	(Z)			0.0300
171.4	0.352				0.0697
Weighted					0.0284
361.0	0.982	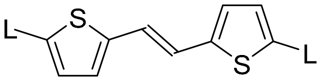	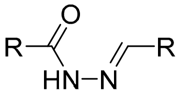	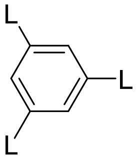	0.0072
245.5	0.769				0.1397
193.4	0.630				0.0513
278.9	0.568	(S)			0.1751
184.9	0.444				0.1529
Weighted					0.0595
361.1	0.987	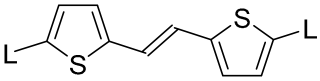	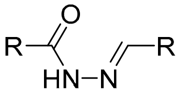	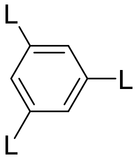	0.0082
273.0	0.642				0.1581
192.6	0.596				0.1063
183.6	0.501	(Z)			0.1388
239.2	0.417				0.1434
Weighted					0.0532
393.3	0.990	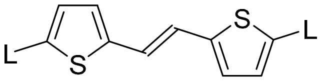	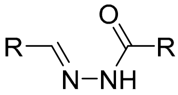	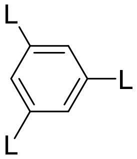	−0.0169
232.2	0.502				−0.0201
266.1	0.344				−0.1418
208.8	0.326	(S)			0.1138
181.8	0.298				−0.1240
Weighted					−0.0352
394.8	0.986	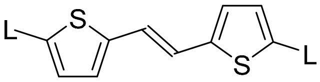	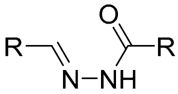	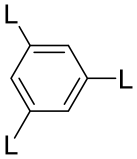	−0.0143
231.9	0.522				−0.0140
207.3	0.412				−0.1413
181.4	0.332	(Z)			−0.2025
251.6	0.325				−0.2867
Weighted					−0.0515
348.2	0.981	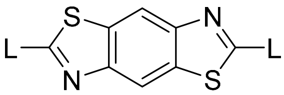	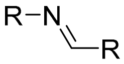	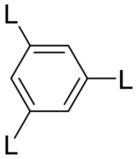	−0.0882
236.7	0.696				0.0764
210.5	0.478				−0.1142
294.5	0.471				−0.0461
204.2	0.444	(S)			−0.1148

Weighted					−0.0990
344.4	0.987	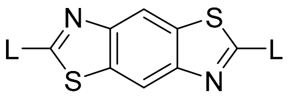 (Z)	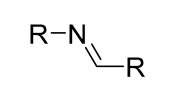	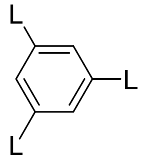	−0.0888
244.3	0.692				0.0672
211.0	0.507				−0.1008
204.3	0.483				−0.1673
175.2	0.466				−0.0893
Weighted					−0.0717
346.6	0.929	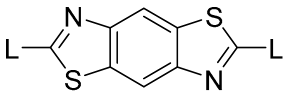 (S)	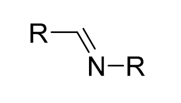	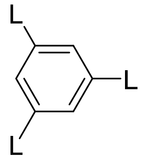	0.1225
304.0	0.796				−0.0132
176.9	0.745				−0.1586
240.5	0.696				0.1650
194.5	0.637				−0.1004
Weighted					0.0336
339.6	0.954	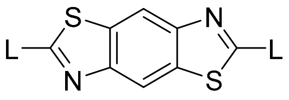 (Z)	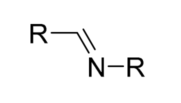	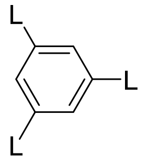	0.0935
174.1	0.728				−0.1648
219.3	0.720				0.0137
191.7	0.644				−0.1964
236.8	0.617				0.1484
Weighted					−0.0187
296.3	0.914	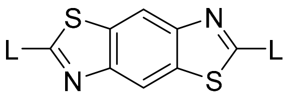 (S)	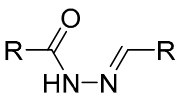	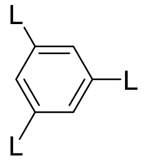	0.0388
288.6	0.825				0.0340
235.7	0.744				0.1914
213.2	0.610				0.0065
190.9	0.588				0.1435
Weighted					0.0505
313.2	0.934	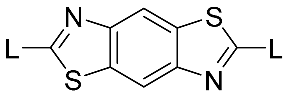 (Z)	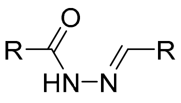	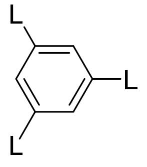	0.0543
305.6	0.867				0.0303
237.5	0.790				0.1658
192.0	0.710				0.1207
182.6	0.644				−0.0562
Weighted					0.0552
325.6	0.991	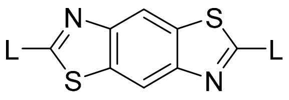 (S)	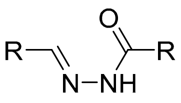	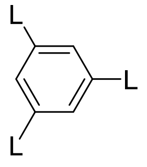	−0.0225
240.7	0.795				−0.0370
217.6	0.626				−0.1890
176.7	0.562				−0.0372
183.6	0.335				−0.2350
Weighted					−0.0646
326.1	0.959	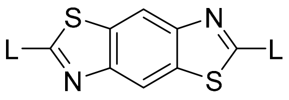 (Z)	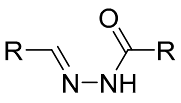	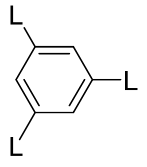	−0.0216
316.1	0.817				−0.0265
236.8	0.666				−0.0621
176.1	0.585				−0.0024
218.2	0.541				−0.1624

Weighted					−0.0716

[1] For certain systems (B02, B06, B07, B08), two near-coplanar conformers resulting from single-bond rotation between the ***UnitB*** and linker are identified by the symbols S and Z.
